# Elimination of CDX2 restricts intestinal hybrid differentiation signatures in stem cell-derived hepatocyte-like cells

**DOI:** 10.1186/s13287-025-04696-6

**Published:** 2025-10-07

**Authors:** Patrick Nell, Antonia Thomitzek, David Feuerborn, Andreas Scholtz-Illigens, Sarah M. Seidel, Katharina Derksen, Lara Maria Chilinski, Kathrin Kattler-Lackes, Nils Blüthgen, Markus Morkel, Jörn Walter, Karolina Edlund, Jörg Rahnenführer, Jan G. Hengstler

**Affiliations:** 1 Department of Toxicology, Leibniz Research Centre for Working Environment and Human, Factors at TU Dortmund, Ardeystraße 67, 44139 Dortmund, Germany; 2 Department of Genetics, University of Saarland, 66123 Saarbrücken, Germany; 3 Institute of Pathology, Charité-Universitätsmedizin Berlin, Freie Universität Berlin and Humboldt-Universität zu Berlin, 10117 Berlin, Germany; 4 Institut Für Biologie, Humboldt-Universität zu Berlin, 10115 Berlin, Germany; 5 Department of Statistics, TU Dortmund University, 44221 Dortmund, Germany

**Keywords:** Hybrid differentiation, Induced pluripotent stem cells, CRISPR, Lineage restriction, Bile canaliculi

## Abstract

**Background and aims:**

The generation of functionally mature hepatocytes from human induced pluripotent stem cells (iPSC) has the potential to replace primary human hepatocytes (PHH) as the gold standard in vitro model for drug screening, studies of hepatotoxicity as well as liver disease, and is considered a gateway technology to future cell therapy applications. However, we recently reported that current protocols for deriving hepatocyte-like cells (HLC) from iPSC fail to restrict differentiation to the hepatic lineage. Instead, single cell transcriptomics and protein expression analysis uncovered that current methods induce hybrid differentiation, leading to the establishment of abundant hepatic and intestinal gene expression signatures within individual HLC, thereby compromising hepatocyte functionality and phenotype.

**Methods:**

Differentiation of iPSC to HLC was performed and followed by analysis of transcriptome changes during iPSC to HLC differentiation in comparison to PHH. Differentiation pattern clustering (DiPaC) of differentially expressed genes (DEG) and downstream bioinformatic analysis identified the gene regulatory networks (GRN) involved in establishing hybrid differentiation signatures in HLC, indicating a major influence of caudal-domain type 2 (CDX2) in HLC-IEC hybrid differentiation. CRISPR Cas9-based genetic engineering was used to generate *CDX2*^*−/−*^ iPSC, followed by HLC differentiation, transcriptomics and intestinal and hepatic protein expression analysis. The observed phenotypic changes in *CDX2*^*−/−*^ HLC were verified by functional analysis of bile canaliculus transport kinetics as well as intestinal enzyme activity and compared to PHH.

**Results and conclusion:**

Transcriptome analysis of WT iPSC to HLC differentiation in comparison to PHH confirmed the establishment of intestinal differentiation gene expression signatures in HLC and indicated a major role of CDX2 in erroneous lineage decision making of HLC. Absence of CDX2 during iPSC to HLC differentiation promoted hepatic specification through the induction of HHEX and PROX1, while drastically reducing intestinal differentiation signatures. *CDX2*^*−/−*^ HLC displayed phenotypic maturation of bile canaliculi, including loss of intestine-associated membrane proteins (e.g. IBAT, SI, LCT), increase in hepatic transporters (e.g. *BSEP*) and size reduction, leading to closer functional and architectural resemblance to bile canaliculi formed by PHH.

**Graphical abstract:**

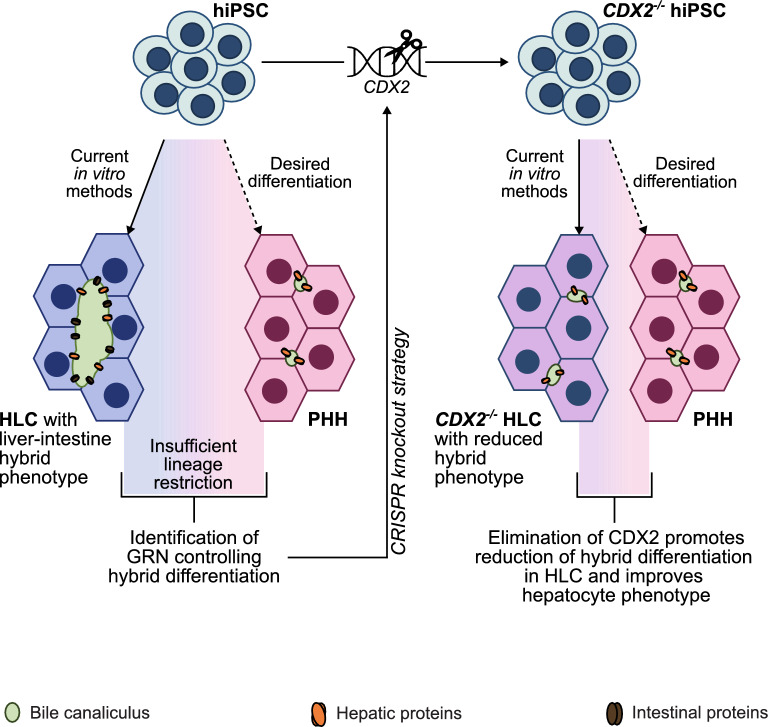

**Supplementary Information:**

The online version contains supplementary material available at 10.1186/s13287-025-04696-6.

## Introduction

In the past two decades, numerous in vitro approaches have been developed to differentiate human induced pluripotent stem cells (iPSC) to hepatocytes [1–6] with the aim to establish an unlimited resource, addressing the scarce availability of primary human hepatocytes (PHH) for research and development of future applications in regenerative medicine, drug screening and toxicology. However, on the single cell level, we recently demonstrated that publicly available and frequently applied protocols for iPSC to hepatocyte differentiation induce a hybrid transcriptome state within individual cells [7]. This hybrid state is characterized by gene regulatory network (GRN) activity of both hepatocytes and intestinal epithelial cells (IEC), thus representing inadequate lineage restriction. Overcoming hybrid differentiation in vitro is of high relevance as it is a pre-requisite for establishing reliable in vitro model systems and increasing safety in future stem cell-based (cell-) therapeutic contexts. Here, we show that caudal-domain type 2 (CDX2) is a critical regulator of the HLC-IEC hybrid state. Its deletion abolished or significantly reduced the expression of IEC-associated genes and simultaneously enhanced hepatocyte differentiation without inducing features of other, non-hepatic definitive endoderm-derived cell types. The latter translated to a functionally improved HLC phenotype with loss of intestinal brush border-associated proteins in bile canalicular membranes and enhanced bile canalicular transport activity.

## Results

### CDX2 is associated with a prominent intestinal phenotype of the WT HLC transcriptome

**Fig. 1:**
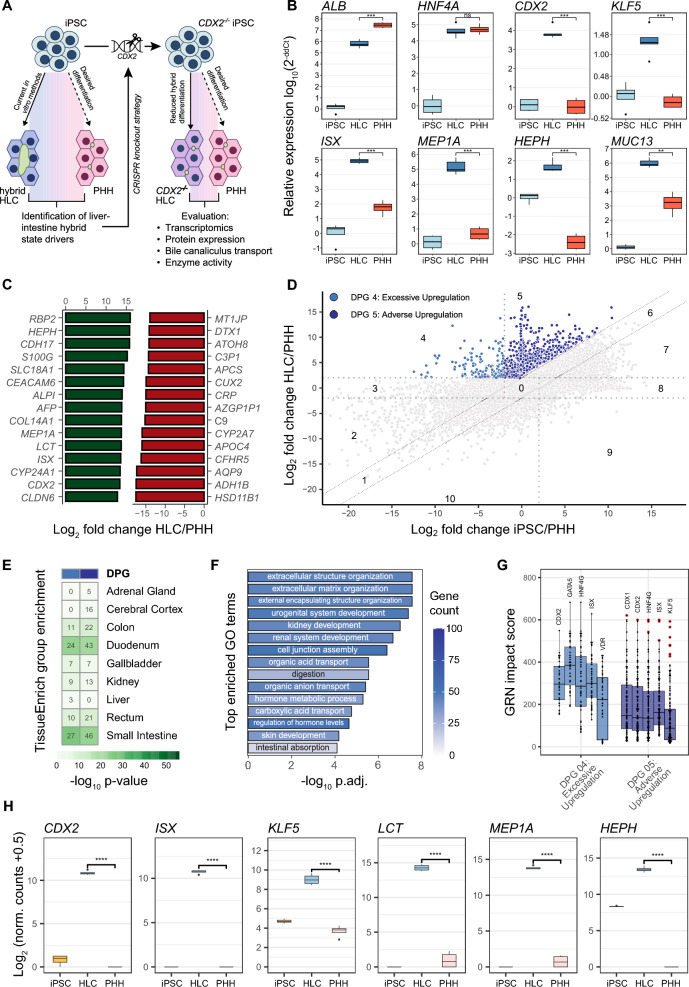
WT HLC transcriptome exhibits prominent intestinal phenotype under control of CDX2. **A** Schematic of the study design. **B** Relative expression of selected markers of hepatic and intestinal differentiation in WT HLC (n = 5) and PHH (n = 4) relative to iPSC (n = 4). **C** Top 15 highest (green) and lowest (red) differentially expressed genes in WT HLC compared to PHH (adj. p-value < 0.001). **D** Supervised logical differentiation pattern clustering (DiPaC) of gene expression trajectories obtained from RNA-sequencing data of iPSC, WT HLC and PHH, visualized in the differentiation pattern plot (DiPa plot). Differentiation pattern groups (DPG) representative of excessively (DPG4) and adversely (DPG5) induced gene upregulation are colored, all other DPG are shown in grey. The x-axis represents log_2_ fold changes of iPSC over PHH, the y-axis indicates log_2_ fold changes of HLC over PHH. Dotted lines represent cut-offs of the clustering approach. **E** Heatmap of tissue group enriched genes in DPG 4 and 5. The color scale indicates the p-values for enriched tissues per DPG; numbers in the heatmap indicate numbers of group enriched genes per tissue and DPG. **F** Bar plot of biological process GO term enrichment in DPG4 and 5 combined. **G** GRN impact scores of target genes (dots) of corresponding transcription factors (boxes) for differential pattern groups 4 and 5. Higher impact scores indicate higher influence of a TF in the corresponding DPG. **H** Normalized count data from RNA sequencing for selected intestinal markers compared between iPSC, HLC and PHH (n = 4 each); levels of significance: *: p =  < 0.05—****: p < 0.0001

To identify gene regulatory networks (GRN) in HLC indicative of differentiation to cell types other than hepatocytes, we first reproduced the previously described HLC hybrid state [7] by differentiating wild type (WT) iPSC (ChiPSC18; Takara Bio) to HLC (Fig. 1A). Indeed, qRT-PCR analysis of hepatocyte (*ALB*, *HNF4A*) and IEC (*CDX2*, *KLF5*, *ISX*, *MEP1A*, *HEPH*, *MUC13*) marker expression in iPSC, HLC and PHH confirmed that HLC acquired a prominent HLC-IEC hybrid phenotype (Fig. 1B). On the genome wide scale, RNA-sequencing of WT iPSC, HLC and PHH revealed that genes associated with intestinal differentiation, including *HEPH*, *MEP1A*, *LCT*, *ISX* and *CDX2*, were among the 15 most upregulated differentially expressed genes (DEG) in HLC compared to PHH (Fig. 1C). Next, supervised differentiation pattern clustering (DiPaC) of DEG was applied (Fig. 1D), allowing us to discriminate differentiation pattern groups (DPG) of genes whose expression levels become more similar to those of PHH during differentiation, such as DPG2, 3, 7 and 8, from groups of genes whose expression levels become less similar to that of PHH (DPG4, 5, 9, 10). We then focused on DPG4 and 5, because the expression of these genes was induced by the differentiation process, although their expression levels should have decreased to resemble those of PHH. As expected, DPG4 and 5 were significantly enriched with genes representative of intestinal tissues such as duodenum, small intestine and to a lesser extent colon, rectum, kidney and gallbladder (Fig. 1E). The intestinal properties of DPG4 and 5 were reflected in enriched gene ontology (GO) terms such as digestion (GO:0007586; p-value < 0.001) and intestinal absorption (GO:0050892; p-value < 0.001; Fig. 1F). Interestingly, CDX2 was prominent among the top five enriched transcription factors in DPG4 and 5 as evidenced by the GRN impact score (Fig. 1G), in line with the established role of CDX2 during embryonic patterning of the developing gut tube. High expression of the intestinal marker genes *CDX2*, *ISX*, *KLF5*, *LCT*, *MEP1A* and *HEPH* in HLC compared to iPSC, PHH (Fig. 1H) and normal colon tissue (Figure S1) confirmed these observations, although it should be noted that IECs from various regions of the gastrointestinal tract will differ in their expression profile of intestinal marker genes and normal colon tissue contained cell types other than IEC. DEG, DiPaC and downstream analysis results are available in Dataset S1. Importantly, we furthermore confirmed that the HLC-IEC hybrid state occurred in several published 2D hepatocyte [8] and 3D human liver organoid [6] differentiation protocols, all of which induced CDX2 and CDX2-dependent gene expression, demonstrating that HLC-IEC hybrid differentiation represents a widespread problem that has not yet been overcome (Figure S2A-D, Dataset S2).

### CDX2 knockout decreased intestinal and promoted expression of hepatic specification markers

**Fig. 2:**
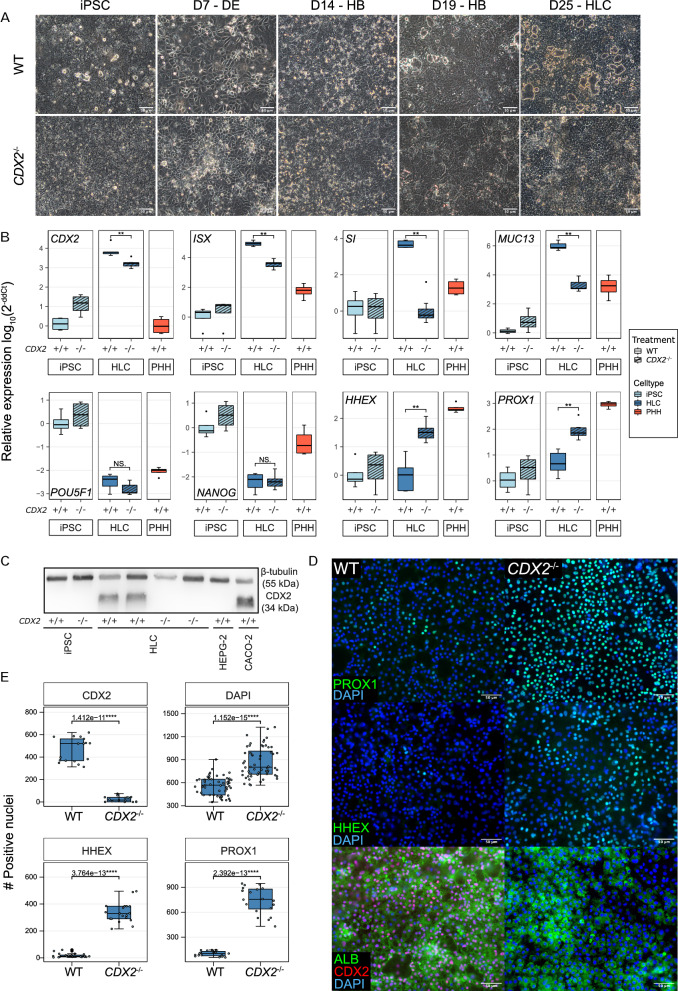
*CDX2*^−/−^ HLC display decreased intestinal and enhanced hepatic marker expression. **A** Side by side comparison of phase contrast images of WT and *CDX2*^−/−^ HLC morphology and cell culture appearance at various differentiation stages. iPSC: day 0 induced pluripotent stem cells; D7-DE: day 7 definitive endoderm; D14-HB: day 14 hepatoblast; D19-HB: day 19 hepatoblast; D25-HLC: day 25 hepatocyte-like cells. **B** qRT-PCR data of markers of intestinal differentiation in *CDX2*^−/−^ iPSC, WT and *CDX2*^*−/−*^ HLC and PHH compared to WT iPSC. Error bars represent standard deviation of n = 4 biological replicates. **C** Western blot against CDX2 and beta-tubulin for WT and *CDX2*^−/−^ iPSC and HLC and additional samples from the colorectal cancer cell line CACO-2 and hepatocellular carcinoma cell line HEPG2. **D** Fluorescence imaging of immunocytochemical stainings of PROX1 (green), HHEX (green), ALB (green), CDX2 (red) and DAPI (blue) in WT HLC and *CDX2*^−/−^ HLC at day 25 of differentiation. **E** Quantification of positive nuclei in immunocytochemical stainings of WT and *CDX2*^−/−^ against CDX2, DAPI, HHEX and PROX1 (n = 3; quantification from at least 5 fields of view each; t-test for statistical significance; *: p =  < 0.05—****: p < 0.0001)

Having identified CDX2 as a potential driver of HLC-IEC hybrid differentiation, we continued to study the role of CDX2 in transcriptional regulation during HLC differentiation. First, a *CDX2*^−/−^ iPSC clone of the ChiPSC18 cell line was generated using sgRNA-based Cas9 targeting to exon 2 of the *CDX2* coding sequence (Figure S3A-C) and validated using enzymatic heteroduplex cleavage detection, DNA sequencing and Western blotting (Figure S3D-F). Next, WT and *CDX2*^−/−^ iPSC were differentiated to WT and *CDX2*^−/−^ HLC, respectively, to verify that both cell lines were competent to form definitive endoderm (DE) and HLC populations. Morphologically, WT and *CDX2*^−/−^ cells were indistinguishable in the iPSC (day 0), definitive endoderm (day 7), and hepatoblast stage (day 14). By day 25, structures resembling bile canaliculi of various sizes were formed between WT cells, whereby some diameters were much larger (8.13 ± 4.91 µm) compared to bile canaliculi in vivo (0.5–3 µm) [9, 10]. In contrast, *CDX2*^−/−^ HLC formed much smaller bile canaliculi with homogeneous size distribution (Fig. 2A).

Notably, the mRNA expression of *CDX2* was reduced but not abolished in *CDX2*^−/−^ compared to WT HLC (Fig. 2B), because the frameshift introduced in exon 2 of the *CDX2* coding sequence did still allow for detection of transcripts by qRT-PCR. Yet, the mRNA levels of the IEC-associated transcription factors *ISX* (Fig. 2B) and *KLF5* (Figure S4A) decreased significantly, accompanied by a drastic reduction of the IEC maturation markers *SI* and *MUC13* (Fig. 2B) to or below the expression levels in PHH. Both WT and *CDX2*^−/−^ HLC exhibited a similar reduction in mRNA expression of the pluripotency-associated transcription factors *POU5F1* and *NANOG* compared to WT iPSC (Fig. 2B), as well as upregulation of hepatocyte markers *ALB* and *HNF4A* on day 25 (Figure S4A). Moreover, transcripts encoding *HHEX* and *PROX1*, both transcription factors of importance in hepatocyte lineage determination [11, 12] and maturation [13, 14], were upregulated from more than 100-fold lower expression in WT HLC to less than tenfold lower expression in *CDX2*^−/−^ HLC compared to PHH (Fig. 2B).

Western blot (WB) analysis confirmed the absence of CDX2 protein in PHH, hepatocellular carcinoma cell line (HepG2), iPSC and *CDX2*^−/−^ HLC, while prominent CDX2 expression was observed in WT HLC and colorectal carcinoma cell line (Caco-2) (Fig. 2C, Figure S5). Immunofluorescence imaging and quantification of CDX2, HHEX and PROX1 in WT and *CDX2*^−/−^ HLC supported the mRNA and WB data, also revealing a higher cell density in *CDX2*^−/−^ HLC as evidenced by the number of DAPI positive nuclei per area (Fig. 2D, E; Dataset S3). This was in line with the observed phenotypic difference, where large areas in WT HLC were covered by bile canaliculus-like, luminar structures (Fig. 2A).

Interestingly, in contrast to *CDX2*^*−/−*^ HLC, mRNA levels of intestinal markers *CDX2*, *KLF5* and *SI* in HLC derived from a heterozygous *CDX2* knockout iPSC clone were not significantly altered compared to WT HLC, with the exception of *HEPH*, even though comparably weak CDX2 protein expression was observed in immunostainings of CDX2 and AFP (Figure S6).

### CDX2^−/−^ HLC exhibit genome-wide reduction of hybrid differentiation

**Fig. 3:**
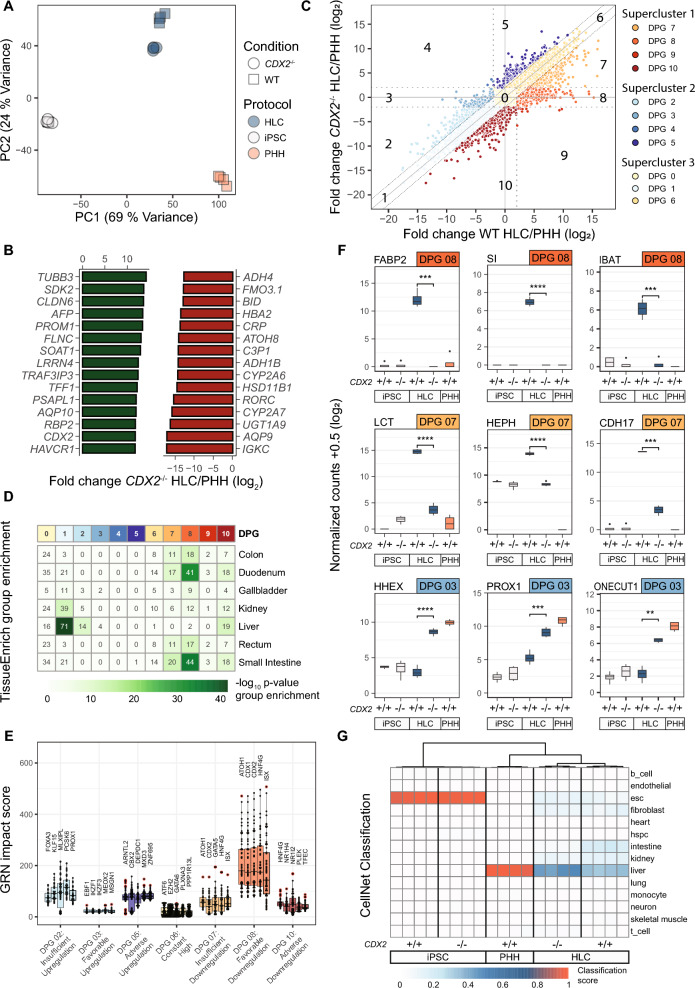
*CDX2*^−/−^ causes genome wide reduction of HLC hybrid state. **A** PCA of the 1,000 most variable genes between iPSC (WT/ *CDX2*^−/−^), HLC (WT/ *CDX2*^−/−^) and PHH obtained by RNA-sequencing (all n = 4). **B** Differential genes with the highest absolute log_2_ fold changes and FDR-adjusted p-values < 0.001 for *CDX2*^−/−^ HLC against PHH. **C** Supervised logical clustering of intervention results obtained from RNA sequencing data according to the DiPaC procedure. The x-axis represents log_2_ fold changes of WT HLC over PHH, the y-axis indicates log_2_ fold changes of *CDX2*^−/−^ HLC over PHH. Dotted lines represent cut-offs of the clustering approach. **D** Heatmap of tissue group gene enrichment across all differentiation pattern groups (DPG). The color scale indicates the -log_10_ p-values for enriched tissues per DPG; numbers in the heatmap indicate numbers of group enriched genes per tissue and DPG. **E** GRN impact score for selected differentiation pattern groups. Higher impact scores indicate higher influence of a TF in the corresponding DPG. **F** Normalized count data from RNA sequencing for selected intestinal and hepatic markers compared between WT and *CDX2*^−/−^ iPSC, HLC and PHH. **G** CellNet classification score of RNA-sequencing data of the indicated cell populations for several reference tissue identities. Levels of significance: *: p =  < 0.05—****: p < 0.0001

Next, we analyzed RNA-seq data from WT and *CDX2*^−/−^ HLC, iPSC and PHH to evaluate the transcriptome-wide impact of CDX2 as a regulator of HLC-IEC hybrid differentiation. Principal component analysis (PCA) (Fig. 3A) indicated a clear discrimination of iPSC, HLC and PHH clusters, with WT and *CDX2*^−/−^ HLC situated closer to PHH than iPSC along the first principal component (PC1, 69% variance). PC2 (24%) on the other hand segregated WT and *CDX2*^−/−^ HLC from iPSC and PHH, illustrating the contribution of hybrid differentiation to the HLC phenotype. Remarkably, *CDX2*^−/−^ HLC displayed a noticeable shift from WT HLC along PC2, approaching iPSC and PHH, thus indicating reduced hybrid differentiation. In contrast to the previous observations among the top DEG in WT HLC compared to PHH (Fig. 1C; Dataset S1), we observed a reduction in intestinal differentiation associated genes examining the top 15 DEG in *CDX2*^−/−^ HLC compared to PHH (Fig. 3B; Dataset S4), but still observed *CDX2* mRNA expression, which we confirmed to not lead to successful translation (Fig. 2C-D).

To evaluate the full spectrum of gene expression changes between WT and *CDX2*^−/−^ HLC, DEG were clustered into differentiation pattern groups (DPG) by supervised DiPaC (Fig. 3C) according to their expression change from WT to *CDX2*^−/−^ HLC in comparison to PHH. The resulting DPG were categorized into three superclusters: SC1) Downregulation in *CDX2*^−/−^ HLC compared to WT HLC (DPG7-10; 1,043 genes), SC2) Upregulation in *CDX2*^−/−^ HLC compared to WT HLC (DPG2-5; 697 genes) and SC3) No change in *CDX2*^−/−^ HLC compared to WT HLC (DPG0, 1 and 6; 18,450 genes). Importantly, within SC1, we found a significant difference (binomial test p-value < 2.2e−16) in the number of genes that were downregulated in *CDX2*^−/−^ HLC compared to WT HLC in favor of reaching PHH expression levels (DPG7 and 8; 690 genes) compared to genes that were downregulated excessively or adversely to reaching PHH levels (DPG9 and 10, respectively; 353 genes), demonstrating that elimination of CDX2 resulted in overall more PHH-like expression of downregulated genes.

In SC1 and 2, DPG8 was the largest group (453 genes), containing genes downregulated to expression levels close to PHH and thus reflecting successfully reduced hybrid differentiation after *CDX2* knockout. It was characterized by an overrepresentation of genes associated with intestinal cell types (Fig. 3D) and transcriptional regulation by HNF4G, ATOH1, ISX, CDX1 and CDX2 (p-value < 0.001; Fig. 3E). Accordingly, DPG8 contained marker genes of IEC differentiation and maturation, including the intestinal type fatty acid binding protein (*FABP2*), sucrase isomaltase (*SI*), as well as the ileal sodium and bile acid transporter *IBAT* (*SLC10A2*) (Fig. 3F). In line with these observations, DPG8 was significantly enriched with genes associated with intestinal metabolism related GO terms, including digestion (GO:0007586; p-value < 0.001; Dataset S4). Interestingly, among the most significantly overrepresented GO terms we found processes reflecting architectural changes of tube structures (GO:0035150; GO:0035296; p-value < 0.001) (Figure S4B), which may be related to the observed phenotypic maturation of bile canaliculus-like structures in *CDX2*^−/−^ HLC. The elimination of intestinal hybrid differentiation-associated gene expression represented by DPG8 was accompanied by a less pronounced reduction of hybrid gene expression in DPG7 (237 genes), referring to genes that were significantly downregulated in *CDX2*^−/−^ HLC but remained above PHH expression levels (Fig. 3C). Similar to DPG8, this group was significantly enriched with genes that are representative of small intestine and duodenum epithelial cells (Fig. 3D), such as lactase (*LCT*), hephaestin (*HEPH*) and cadherin 17 (*CDH17*) (Fig. 3F), and predominantly regulated by CDX2, ISX and HNF4G (p-value < 0.001, Fig. 3E). Accordingly, CDX2-associated genes were significantly enriched among DPG7 and DPG8 (odds ratio = 27.81, Fisher’s exact test p = 9.34 × 10^–84^; according to Enrichr-based transcription factor overrepresentation analysis; Dataset S4), indicating highly specific downregulation of CDX2-associated genes following CDX2 knockout.

Finally, the observed reduction of hybrid gene expression came at the cost of adverse downregulation of certain genes (DPG10; 342 genes; Fig. 3C), which were not only linked to gut, but also liver tissue (Fig. 3D). Here, the overlap of intestine and liver-enriched genes was at least 50% (Figure S4C). However, transcriptional regulation of genes in DPG10 was shown to be enriched for HNF4G, NR1H4 and NR1I2, rather than CDX2, with overall comparably low GRN impact scores (Fig. 3E; Dataset S4). A potential explanation for this observed downregulation could be the well described role of CDX2 in the activation of HNF4 during fetal development of the gastrointestinal tract [15], hinting at a deficit in liver-specific HNF4 recruitment during in vitro HLC differentiation. Thus, observations from SC1 suggest that *CDX2*^−/−^ HLC benefited from a drastic reduction in IEC-associated hybrid differentiation programs at the cost of a significantly smaller number of genes that were downregulated below PHH expression levels. In support of the preceding analysis, CellNet, an independent, objective network biology approach for classification of the cellular differentiation states [16], confirmed that *CDX2*^−/−^ HLC acquired almost no intestinal differentiation state and, on the contrary, indicated a minor increase of hepatic identity in *CDX2*^−/−^ HLC compared to WT (Fig. 3G).

### CDX2 knockout favors hepatic specification without promoting non-hepatic phenotypes

Having demonstrated that *CDX2*^−/−^ HLC acquired almost no intestinal differentiation state, we focused on SC2, which contained all genes that were upregulated during *CDX2*^−/−^ HLC compared to WT HLC differentiation. In contrast to SC1, there was no significant difference in the number of genes in SC2 that were upregulated in favor of reaching PHH expression levels (DPG2 and 3; 351 genes) compared to those being excessively or adversely upregulated (DPG4 and 5, respectively; 346 genes). While the number of tissue group enriched liver genes in DPG3 (171 genes) did not indicate a significant improvement of the liver phenotype in *CDX2*^−/−^ HLC (Fig. 3D), we found that expression levels of the transcription factors *PROX1*, *HHEX* and *ONECUT1* were remarkably increased in *CDX2*^−/−^ HLC compared to WT (Figs. 2E, D, 3F). Consequently, transcriptional regulation by PROX1 was also found to be enriched in DPG2 (180 genes) (p-value < 0.001; Fig. 3E). Moreover, *CDX2* knockout did not lead to notably excessive induction of genes beyond expression levels of PHH (DPG4; 3 genes). Nevertheless, it did induce upregulation of genes whose expression should have remained unaltered or even been downregulated to reach PHH expression levels (DPG5; 343 genes). This group of genes was especially important for evaluating the success of the *CDX2* knockout with regard to not inducing additional hybrid differentiation. However, there was no significant overrepresentation of genes enriched in other endoderm-derived cell types in DPG5 (Fig. 3D). Rather, overrepresented GO terms and transcription factors suggested that the majority of induced genes were associated with the regulation of cell proliferation (e.g. nuclear division; GO:0000280 and MXD3; p-values < 0.001) and chromatin organization (e.g. CBX2; p-value < 0.001) processes (Fig. 3E; Dataset S4). In accordance, CellNet analysis did not indicate the emergence of additional non-hepatic differentiation signatures in *CDX2*^−/−^ HLC (Fig. 3G). The full list of DEGs and enrichment analysis results is available in Dataset S4.

### CDX2^−/−^ HLC lose IEC-associated protein expression and function and display an improved bile canalicular phenotype

**Fig. 4:**
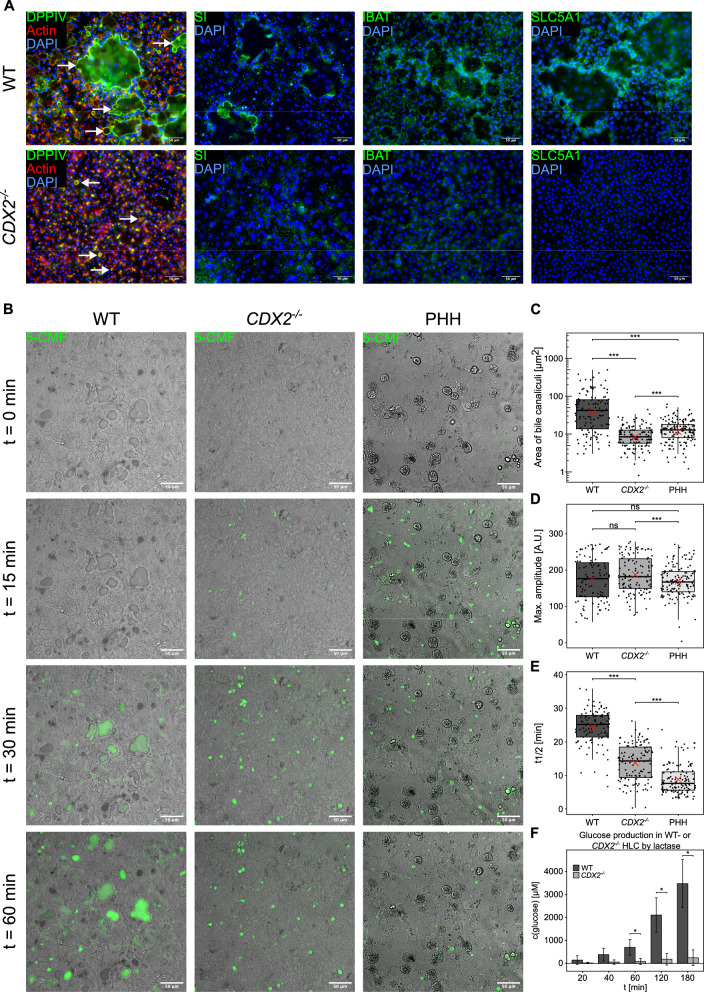
*CDX2*^−/−^ HLC display an improved bile canalicular phenotype. **A** Fluorescence imaging of immunocytochemical stainings with antibodies against DPPIV (green), SI (green), IBAT (green), SLC5A1 (green), and rhodamine-phalloidin against f-actin (red) and DAPI (blue) as nuclear stain in WT and *CDX2*^−/−^. White arrows indicate bile canaliculus-like structures. **B** Confocal laser scanning microscopy images showing the secretion of green fluorescent 5-CMF over 60 min by WT, *CDX2*^−/−^ HLC and PHH into bile canaliculi after exposure to CMFDA. **C** Quantification of the area of bile canaliculi in WT, *CDX2*^−/−^ HLC and PHH, each dot representing an individual bile canaliculus. **D** Quantification of the maximum amplitude and **E** half-time of canalicular fluorescence intensity (n = 3 biological replicates each with 120 observations for WT, 142 for *CDX2*^−/−^ HLC and 150 for PHH). **F** Glucose production by lactase in WT and *CDX2*^−/−^ (n = 3). Levels of significance: *: p =  < 0.05—****: p < 0.0001

After evaluating the transcriptome-wide effects of *CDX2* knockout on hepatocyte differentiation, we investigated whether selected RNA expression alterations translated to the functional protein level. Immunocytochemical staining against DPPIV and f-actin, as well as the IEC markers SI, IBAT and SLC5A1 were performed and revealed prominent signals lining the lumen of bile-canaliculus-like structures of various sizes in WT HLC (Fig. 4A). Similar to PHH, these structures were previously reported to secrete 5-chloromethylfluorescein diacetate (CMFDA) into their lumen [7, 17]. While this indicated the translation of the liver-intestine hybrid transcriptome state to the level of protein expression in WT HLC, the expression of all three IEC markers was abolished in *CDX2*^−/−^ HLC (Fig. 4A). Moreover, *CDX2*^−/−^ HLC displayed a drastically changed bile canaliculus phenotype with a more homogeneous size distribution and hepatocyte-characteristic polarization of surrounding HLC compared to the variable dimensions of bile canaliculus-like structures observed in WT HLC (Fig. 4A, indicated by white arrows). Together with the observation of increased expression of the hepatocyte-specific bile salt export pump (*BSEP*, Figure S4D), this led us to investigate the functionality of bile canaliculi in WT and *CDX2*^−/−^ HLC compared to PHH. Treatment with CMFDA (Fig. 4B) allowed us to quantitatively assess bile canaliculus size distribution (Fig. 4C) and transport kinetics of both observed phenotypes, revealing comparable distribution of signal amplitudes (Fig. 4D), but much faster secretion and PHH-like size distribution in *CDX2*^−/−^ HLC (p-value < 0.001, Fig. 4E; Dataset S5). Additionally, assaying lactase (LCT) activity indicated that glucose production from lactose was drastically reduced (93%) in *CDX2*^−/−^ HLC (Fig. 4F), further supporting the loss of intestinal hybrid properties of the bile canalicular membranes.

## Discussion

Phenotypically stable hepatocytes derived from iPSC will facilitate scientific advancements in liver-targeted research and therapy development. Yet, current methods for differentiation of HLC from human induced pluripotent stem cells (iPSC) fail to establish an exclusive hepatocyte identity and their applications remain limited. The primary challenge to generating fully mature and functional stem cell-derived hepatocytes in vitro lies in overcoming hybrid differentiation—a phenomenon where hepatocyte-like cells (HLC), derived using various protocols, exhibit gene expression profiles not only typical of primary human hepatocytes (PHH), but also of e.g. intestinal epithelial cell (IEC) types. Notably, hybrid differentiation does not produce distinct subpopulations but occurs within individual cells, and manifests as a 'hybrid state' of the HLC transcriptome. Isolated fetal liver cells did not display a similar phenomenon [7], suggesting that the here described hybrid differentiation does not occur in vivo. Although both intestine and liver are derived from the primitive gut tube, which is patterned along the anterior–posterior-axis into foregut, midgut and hindgut, the liver develops from the posterior domain of the foregut, while intestine and colon originate from the mid- and hindgut. This partitioning depends on temporally and spatially defined cues, including WNT, FGF and BMP signaling in the posterior domain of the gut tube and expression of antagonists in the anterior domain leading to concentration gradients along the axis [18]. This results in the establishment of mutually exclusive *SOX2* and *CDX2* expression domains in the foregut, and mid- to hindgut, respectively, marking the stomach-intestine-boundary-line, where hepatic and pancreatic buds form [19]. Failure to reproduce the precise interplay of these developmental cues in vitro likely results in insufficient restriction of cell fates with respect to specific positional identities. To overcome hybrid differentiation in vitro, a comprehensive understanding of the involved GRN landscape and development of molecular interventions are required to restrict cell fate to the hepatocyte lineage, as well as to promote the hepatocyte maturation process. Here, we present a comprehensive transcriptomic analysis of the HLC-IEC hybrid state, confirming the transcription factor caudal domain type 2 (CDX2) as a critical regulator of hybrid differentiation that leads to the induction of IEC marker gene expression at levels comparable to normal colonic tissue, and demonstrated that CRISPR Cas9 mediated knockout of *CDX2* in iPSC enabled the production of *CDX2*^−/−^ HLC with a drastically reduced hybrid phenotype. Abolishing *CDX2* expression widely prohibited the establishment of IEC differentiation signatures, resulting in efficient reduction of markers of intestinal functions, such as SI and IBAT, to PHH levels and elimination of IEC-characteristic enzymatic activity of lactase in bile canalicular membranes. Interestingly, HLC derived from heterozygous *CDX2* knockout iPSC acquired a hybrid state comparable to WT HLC, highlighting the requirement of either a complete deletion or substantial suppression of *CDX2* expression for successful elimination of CDX2-dependent hybrid differentiation. We further showed that *CDX2* knockout enhanced hepatocyte lineage specification through HHEX and PROX1 GRN induction, possibly a consequence of lifting CDX2-dependent transcriptional repression, and improved HLC bile canaliculus phenotype and functionality. Intriguingly, there is no evidence of direct interaction between CDX2 and *HHEX* or *PROX1* gene loci. CDX2-dependent repression of the foregut endoderm fate may be achieved through epigenetic mechanisms, such as the modulation of polycomb repressive complex 2 (PRC2) expression and deposition of associated histone modifications, as previously described for the developing yolk sac [20]. Accordingly, knockout of *CDX2* may prevent the transcriptional repression of genes associated with foregut fate establishment, thereby favoring lineage decisions to a hepatic phenotype. Following *CDX2* knockout, we detected a minor increase in hepatic identity of *CDX2*^*−/−*^ HLC by employing CellNet [16] analysis, which is based on an algorithm that objectively assesses stem cell differentiation in comparison to training sets representative of different cell lineages and tissues. Importantly, we did not observe any additional induction of tissue-specific features of non-hepatic cell types following *CDX2* knockout. However, with the exception of functional and phenotypic improvements of bile canaliculus transport kinetics and morphology in *CDX2*^*−/−*^ HLC, which is favored by the reduction in intestinal differentiation, transcriptome changes did not indicate improvements of other hepatocyte functions. Nevertheless, with CDX2 being merely one of multiple key regulators driving hybrid differentiation in HLC, these results indicate that GRN alterations aiming for the restriction of non-hepatocyte lineage differentiation may indirectly promote hepatocyte specification and underline the promising prospect of combining GRN interventions for lineage restriction with those that aim to activate hepatocyte-specific GRN. In theory, titrating a critical set of GRN interventions should allow for a complete elimination of HLC hybrid features and full hepatocyte lineage commitment. Likewise, minimizing the invasiveness of CDX2 depletion strategies, preferably by employing small molecule interventions to modulate transcription factor activity, developmental signaling gradients (e.g. WNT, FGF and BMP) or epigenetic modifications could contribute to a more precise specialization of differentiating cells into posterior foregut endoderm and mitigate hybrid differentiation. Such approaches should then be compared to knockdown experiments targeting CDX2, which are thought to be more prone to inducing additional heterogeneity if not performed in monoclonal cell lines, to evaluate the feasibility of both strategies.

Irrespective of intestinal hybrid differentiation being a general phenomenon among HLC produced by various published methods and the presented strategy showing highly specific effects on the CDX2 GRN, the extent to which GRN manipulations can effectively restrict hybrid differentiation may be subject to clonal and cell line variations, as well as off-target effects. In previous work, we reported that FXR GRN activation could promote hepatocyte maturation and functionality in HLC produced from multiple cell lines and using different published protocols, albeit differences were observed in the efficacy of the treatments. Such limitations are expected to apply to different *CDX2*^*−/−*^ iPSC lines as well. Therefore, it will be important to account for variation among subclones, cell lines and off-target effects, depending on the type of treatment, before proceeding to routine application of GRN optimized HLC in vitro or in therapeutic contexts. Nevertheless, we believe that following this line of research holds promise to identify and finally overcome the roadblocks currently limiting the generation of bona fide hepatocytes from pluripotent stem cells.

## Methods

Human iPSC were cultured in the DEF-CS 500 System (Takara Bio) according to manufacturer’s instructions. *CDX2* knock-out was achieved by targeting exon 2 of the *CDX2* gene in a single guide RNA based CRISPR Cas9 approach. Therefore, a vector encoding Cas9, sgRNA and GFP was transfected into ChiPSC18 cells. Single, GFP-positive cells were clonally expanded and analyzed for deletions in the *CDX2* target region using enzymatic cleavage detection and DNA sequencing. WT-iPSC as well as *CDX2*^±^ and *CDX2*^*−/−*^ iPSC were differentiated towards hepatocyte-like cells (HLC) according to previously published methods [7]. HLC were analyzed by qPCR, Western blot, immunofluorescence staining and RNA sequencing and compared to iPSC and PHH for phenotype evaluation. Lactase activity of WT- and *CDX2*^*−/−*^ HLC was analyzed by measuring glucose production following lactose exposure. HLC and PHH were treated with 5-chloromethylfluorescein diacetate (CMFDA) to quantify fluorescent CMF accumulation in bile canalicular-like structures based on time series captures by confocal laser scanning microscopy. For material and method details, please refer to the supplementary information appendix (SI appendix).

## Supplementary Information

Additional file 1.

Additional file 2.

Additional file 3.

Additional file 4.

Additional file 5.

Additional file 6.

Additional file 7.

## Data Availability

RNA-sequencing data generated for this study was uploaded to NCBI under accession PRJNA1226404. RNA sequencing data of normal human colon tissue is available under accession EGAC00001001472 at the European Genome-Phenome Archive (EGA).

## Abbreviations

iPSC: Induced pluripotent stem cells
PHH: Primary human hepatocytes
CDX2: Caudal domain type 2
GRN: Gene regulatory network
IEC: Intestinal epithelial cells
WT: Wild-type
DEG: Differentially expressed genes
DiPaC: Differentiation pattern clustering
DPG: Differentiation pattern group
WB: Western blot
GO: Gene ontology
SC: Supercluster
CMFDA: 5-Chloromethylfluorescein diacetate
LCT: Lactase
HepH: Hephaestin
CDH17: Cadherin 17
IBAT: Intestinal bile acid transporter
BSEP: Bile salt export pump
FABP2: Intestinal type fatty acid binding protein
SI: Sucrase isomaltase
