# Supplementary material for: Elimination of CDX2 restricts intestinal hybrid differentiation signatures in stem cell-derived hepatocyte-like cells
Source: Stem Cell Res Ther. 2025 Oct 7;16:543. doi: 10.1186/s13287-025-04696-6 (PMC12505559; doi:10.1186/s13287-025-04696-6)
Supplement: Supplementary file 1 — Additional file 1. [file 13287_2025_4696_MOESM1_ESM.docx]

**Supporting Information for**

Elimination of CDX2 restricts intestinal hybrid differentiation signatures in stem cell-derived hepatocyte-like cells

Patrick Nell^1§^, Antonia Thomitzek^1§^, David Feuerborn^1^, Andreas Scholtz-Illigens^1^, Sarah M. Seidel^1^, Katharina Derksen^1^, Lara Maria Chilinski^1^, Kathrin Kattler-Lackes^2^, Nils Blüthgen^3,4^, Markus Morkel^3^, Jörn Walter^2^, Karolina Edlund^1^, Jörg Rahnenführer^5^, Jan G. Hengstler^1^

Patrick Nell

Email: [nell@ifado.de](mailto:nell@ifado.de)

**This file includes:**

Supporting Materials and Methods

Figures S1 to S6

Legends for Datasets S1 to S6

SI References

**Other supporting materials for this manuscript include the following:**

Datasets S1 to S6

Supporting Information Text

Materials

| **REAGENT or RESOURCE** | **SOURCE** | **IDENTIFIER** |
| --- | --- | --- |
| **Antibodies** | | |
| Alexa Fluor® 488 Donkey anti-Goat | Jackson Immuno Research Laboratories | 705-545-003 |
| Alexa Fluor® 488 Donkey anti-Mouse | Jackson Immuno Research Laboratories | 715-545-150 |
| Alexa Fluor® 488 Donkey anti-Rabbit | Jackson Immuno Research Laboratories | 711-545-152 |
| anti-rabbit-HRP | Cell Signaling Technologies | Cat. No.: 7074 |
| Cy3AffiniPure Donkey anti-Rabbit | Jackson Immuno Research Laboratories | 711-165-152 |
| Goat polyclonal-DPPIV (1:100) | R&D Systems | AF954 |
| Mouse monoclonal anti-ALB (1:100) | R&D Systems | MAB1455 |
| Mouse monoclonal anti-SSEA4 (1:400) | Cell Signaling Technologies | 9656 |
| Rabbit monoclonal anti-CDX2 (1:1000) | abcam | ab76541 |
| Rabbit monoclonal anti-HHEX (1:100) | R&D Systems | MAB83771 |
| Rabbit monoclonal anti-NANOG (1:400) | Cell Signaling Technologies | 9656 |
| Rabbit monoclonal anti-OCT4 (1:400) | Cell Signaling Technologies | 9656 |
| Rabbit monoclonal anti-PROX1 (1:500) | abcam | ab199359 |
| Rabbit polyclonal anti-ALB (1:200) | Sigma | HPA031024 |
| Rabbit polyclonal anti-CDX2 (1:400) | abcam | ab227201 |
| Rabbit polyclonal anti-IBAT (1:100) | Atlas Antibodies | HPA004795 |
| Rabbit polyclonal anti-SI (1:100) | Atlas Antibodies | HPA011897 |
| Rabbit polyclonal anti-SLC5A1 (1:100) | Atlas Antibodies | HPA051805 |
| **Biological samples** | | |
| Colon Tissue | Institute of Pathology, Charité-Universitätsmedizin | Donor 1 |
| Colon Tissue | Institute of Pathology, Charité-Universitätsmedizin | Donor2 |
| Colon Tissue | Institute of Pathology, Charité-Universitätsmedizin | Donor 3 |
| Colon Tissue | Institute of Pathology, Charité-Universitätsmedizin | Donor 4 |
| PHH | BioIVT | Donor1 |
| PHH | BioIVT | Donor2 |
| PHH | BioIVT | Donor3 |
| PHH | BioIVT | Donor4 |
| **Chemicals, peptides, and recombinant proteins** | | |
| Agarose | Carl Roth | Cat. No.: 2267.3 |
| Biolaminin 111 LN (LN111) | Biolamina | Cat. No.: LN111-0501 |
| Biolaminin 521 LN (LN521) | Biolamina | Cat. No.: LN521-05 |
| CellTracker™ Green CMFDA | ThermoFisher Scientific | Cat. No.: C2925 |
| Phalloidin-Rhodamine | ThermoFisher Scientific | Cat. No.: R415 |
| UltraPure™ Ethidium bromide, 10 mg/ml | ThermoFisher Scientific | Cat. No.: 15585011 |
| **Critical commercial assays** | | |
| AMPure XP Beads | Beckman Coulter | Cat. No.: A63881 |
| cDNA Reverse Transcription Kit | ThermoFisher Scientific | Cat. No.: 4368814 |
| Cellartis® DEF-CS 500^TM^ culture system | Takara Bio Europe | Cat. No. Y30010 |
| Cellartis® Definitive Endoderm Differentiation Kit | Takara Bio Europe | Cat. No.: Y30035 |
| Cellartis® Hepatocyte Differentiation Kit | Takara Bio Europe | Cat. No.: Y30050 |
| Direct-zol RNA MiniPrep Plus Kit | Zymo Research | Cat. No.: R2072 |
| GeneArt® Genomic Cleavage Detection Kit | Life Technologies | Cat. No.: A24372 |
| Lactase Activity Assay Kit | Elabscience | Cat. No.: E-BC-K131-M |
| Nextera DNA Library Prep Kit | Illumina | Cat. No.: FC-121-1031 |
| NextSeq 500 High Output v2.5 (150 cycles) Kit | Illumina | Cat. No.: 20024907 |
| P3 Primary Cell 4D-Nucleofector® X Kit S | LONZA | Cat. No.: V4XP-3032 |
| Pierce™ BCA Protein Assay Kit | ThermoFisher Scientific | Cat. No.: 23225 |
| RNeasy® Mini Kit | Qiagen | Cat. No.: 74104 |
| TruSeq® SBS Kit v3 | Illumina | Cat. No.: FC-401-3001 |
| TruSeq® Stranded mRNA Library Prep Kit | Illumina | Cat.No.: 20020595 |
| TaqMan Assay *ALB* | ThermoFisher Scientific | Hs00609411_m1 |
| TaqMan Assay *CDX2* | ThermoFisher Scientific | Hs01078080_m1 |
| TaqMan Assay *GAPDH* | ThermoFisher Scientific | Hs02786624_g1 |
| TaqMan Assay *HEPH* | ThermoFisher Scientific | Hs00953259_m1 |
| TaqMan Assay *HHEX* | ThermoFisher Scientific | Hs00242160_m1 |
| TaqMan Assay *HNF4A* | ThermoFisher Scientific | Hs00230853_m1 |
| TaqMan Assay *ISX* | ThermoFisher Scientific | Hs01368145_m1 |
| TaqMan Assay *KLF5* | ThermoFisher Scientific | Hs00156145_m1 |
| TaqMan Assay *MEP1A* | ThermoFisher Scientific | Hs01574676_m1 |
| TaqMan Assay *MUC13* | ThermoFisher Scientific | Hs00217230_m1 |
| TaqMan Assay *NANOG* | ThermoFisher Scientific | Hs02387400_g1 |
| TaqMan Assay *POU5F1* | ThermoFisher Scientific | Hs04260367_gH |
| TaqMan Assay *PROX1* | ThermoFisher Scientific | Hs00896293_m1 |
| TaqMan Assay *SI* | ThermoFisher Scientific | Hs00356112_m1 |
| **Deposited data** | | |
| Raw and analyzed data | This paper | PRJNA1226404 |
| **Experimental models: Cell lines** | | |
| Human: Caco-2 | ATCC | HTB-37 |
| Human: ChiPSC18 | Takara Bio Europe | Cat. No. Y00300 |
| Human: HepG2 | ATCC | HB-8065 |
| **Oligonucleotides** | | |
| CDX2-fwd-primer (5’-GCAGTTCTCAGCCCTCACTT) | Microsynth | Customary design |
| CDX2-rev-primer (5’-GCATCCTCCTGCTTCAGTCT) | Microsynth | Customary design |
| **Recombinant DNA** | | |
| KO-Vector with hCas9, EGFP and gRNA (5’-CCTCTCAGAGAGCCCCAGCGTGG) | Vector Builder | VB211007-1083juq |
| **Software and algorithms** | | |
| Benchling (for academics) | https://www.benchling.com/academic | https://www.benchling.com/academic |
| ImageJ | Schneider et al., 2012  https://www.nature.com/articles/nmeth.2089 | https://imagej.nih.gov/ij/ |
| R (version 4.4.1) | https://www.r-project.org/ | https://www.r-project.org/ |
| R Studio (version 2024.04.2) | Posit PBC | https://posit.co/ |
| ZEN (black edition) | Carl Zeiss Microscopy |  |
| ZEN (blue edition) | Carl Zeiss Microscopy |  |
| biomaRt | Morgan M (2024). BiocVersion: Set the appropriate version of Bioconductor packages. R package version 3.20.0. | https://bioconductor.org/packages/release/bioc/html/BiocVersion.html |
| clusterProfiler R package (v4.14.4) | S Xu, E Hu, Y Cai, Z Xie, X Luo, L Zhan, W Tang, Q Wang, B Liu, R Wang, W Xie, T Wu, L Xie, G Yu. Using clusterProfiler to characterize multiomics data. Nature Protocols. 2024, 19(11):3292-3320 | https://bioconductor.org/packages/release/bioc/html/clusterProfiler.html |
| DESeq2 R package (v1.46.0) | Love, M.I., Huber, W., Anders, S. Moderated estimation of fold change and dispersion for RNA-seq data with DESeq2 Genome Biology 15(12):550 (2014)  doi:10.1186/s13059-014-0550-8 | https://bioconductor.org/packages/release/bioc/html/DESeq2.html |
| dplyr R package (v1.1.4) | Wickham H, François R, Henry L, Müller K, Vaughan D (2023). dplyr: A Grammar of Data Manipulation. R package version 1.1.4, <https://CRAN.R-project.org/package=dplyr> | https://cran.r-project.org/package=dplyr |
| drc R package (v3.0-1) | Ritz, C., Baty, F., Streibig, J. C., Gerhard, D. (2015) Dose-Response Analysis Using R PLOS ONE, 10(12), e0146021 | https://cran.r-project.org/package=drc |
| enrichR R package (v3.2) | https://github.com/wjawaid/enrichR | https://github.com/wjawaid/enrichR |
| factoextra R package (v1.0.7) | Kassambara A, Mundt F (2020). factoextra: Extract and Visualize the Results of Multivariate Data Analyses. R package version 1.0.7, <https://CRAN.R-project.org/package=factoextra> | https://cran.r-project.org/package=factoextra |
| fs R package (v1.6.4) | Hester J, Wickham H, Csárdi G (2024). fs: Cross-Platform File System Operations Based on 'libuv'. R package version 1.6.5, <https://CRAN.R-project.org/package=fs> | https://cran.r-project.org/package=fs |
| ggfittext R package (v0.10.2) | Wilkins D (2024). ggfittext: Fit Text Inside a Box in 'ggplot2'. R package version 0.10.2, <https://CRAN.R-project.org/package=ggfittext> | https://cran.r-project.org/package=ggfittext |
| ggpattern R package (v1.1.1) | FC M, Davis T, ggplot2 authors (2024). ggpattern: 'ggplot2' Pattern Geoms. R package version 1.1.3, <https://CRAN.R-project.org/package=ggpattern> | https://cran.r-project.org/package=ggpattern |
| ggplot2 R package (v3.5.1) | H. Wickham. ggplot2: Elegant Graphics for Data Analysis. Springer-Verlag New York, 2016.  https://doi.org/10.1007/978-3-319-24277-4 | https://cran.r-project.org/package=ggplot2 |
| ggraph R package (v2.2.1) | Pedersen T (2024). ggraph: An Implementation of Grammar of Graphics for Graphs and Networks. R package version 2.2.1, <https://CRAN.R-project.org/package=ggraph> | https://cran.r-project.org/package=ggraph |
| ggsignif R package (v0.6.4) | Ahlmann-Eltze, C., & Patil, I. (2021). ggsignif: R Package for Displaying Significance Brackets for 'ggplot2'. PsyArxiv. doi:10.31234/osf.io/7awm6 | https://cran.r-project.org/package=ggsignif |
| igraph R package (v2.1.2) | Csardi G, Nepusz T (2006). “The igraph software package for complex network research.” InterJournal, *Complex Systems*, 1695. <https://igraph.org> | https://cran.r-project.org/package=igraph |
| MASS R package (v7.3-61) | Venables, W. N. & Ripley, B. D. (2002) Modern Applied Statistics with S. Fourth Edition. Springer, New York. ISBN0-387-95457-0 | https://cran.r-project.org/package=MASS |
| nlstools R package (v2.1-0) | Florent Baty, Christian Ritz, Sandrine Charles, Martin Brutsche, Jean-Pierre Flandrois, Marie-Laure Delignette-Muller (2015). A Toolbox for Nonlinear Regression in R: The Package nlstools. Journal of Statistical Software, 66(5), 1-21. doi:10.18637/jss.v066.i05 | https://cran.r-project.org/package=nlstools |
| org.Hs.eg.db R package (v3.20.0) | Carlson M (2024). org.Hs.eg.db: Genome wide annotation for Human. R package version 3.20.0. | https://bioconductor.org/packages/release/data/annotation/html/org.Hs.eg.db.html |
| PerformanceAnalytics R package (v2.0.4) | Peterson B (2024). PerformanceAnalytics: Econometric Tools for Performance and Risk Analysis. R package version 2.0.4, <https://CRAN.R-project.org/package=PerformanceAnalytics> | https://cran.r-project.org/package=PerformanceAnalytics |
| pheatmap R package (v1.0.12) | Kolde R (2019). pheatmap: Pretty Heatmaps. R package version 1.0.12, <https://CRAN.R-project.org/package=pheatmap> | https://cran.r-project.org/package=pheatmap |
| purrr R package (v1.0.2 | Wickham H, Henry L (2023). purrr: Functional Programming Tools. R package version 1.0.2, <https://CRAN.R-project.org/package=purrr> | https://cran.r-project.org/package=purrr |
| RColorBrewer R package (v1.1-3) | Neuwirth E (2022). RColorBrewer: ColorBrewer Palettes. R package version 1.1-3, <https://CRAN.R-project.org/package=RColorBrewer> | https://cran.r-project.org/package=RColorBrewer |
| readxl R package (v1.4.3) | Wickham H, Bryan J (2023). readxl: Read Excel Files. R package version 1.4.3, <https://CRAN.R-project.org/package=readxl> | https://cran.r-project.org/package=readxl |
| scales R package (v1.3.0) | Wickham H, Pedersen T, Seidel D (2023). scales: Scale Functions for Visualization. R package version 1.3.0, <https://CRAN.R-project.org/package=scales> | https://cran.r-project.org/package=scales |
| sf R package (v1.0-19) | Pebesma, E., & Bivand, R. (2023). Spatial Data Science: With Applications in R. Chapman and Hall/CRC.  https://doi.org/10.1201/9780429459016 | https://cran.r-project.org/package=sf |
| stringr R package (v1.5.1) | Wickham H (2023). stringr: Simple, Consistent Wrappers for Common String Operations. R package version 1.5.1,  <https://CRAN.R-project.org/package=stringr> | https://cran.r-project.org/package=stringr |
| tidygraph R package (v1.3.1) | Pedersen T (2024). tidygraph: A Tidy API for Graph Manipulation. R package version 1.3.1,  <https://CRAN.R-project.org/package=tidygraph> | https://cran.r-project.org/package=tidygraph |
| tidyr R package (v1.3.1) | Wickham H, Vaughan D, Girlich M (2024). tidyr: Tidy Messy Data. R package version 1.3.1,  <https://CRAN.R-project.org/package=tidyr> | https://cran.r-project.org/package=tidyr |
| tidyverse R package (v2.0.0) | Wickham H, Averick M, Bryan J, Chang W, McGowan LD, François R, Grolemund G, Hayes A, Henry L, Hester J, Kuhn M, Pedersen TL, Miller E, Bache SM, Müller K, Ooms J, Robinson D, Seidel DP, Spinu V, Takahashi K, Vaughan D, Wilke C, Woo K, Yutani H (2019). “Welcome to the tidyverse.” Journal of Open Source Software, *4*(43), 1686. doi:10.21105/joss.01686 | https://cran.r-project.org/package=tidyverse |
| TissueEnrich R package (v1.26.0) | Jain A and Tuteja G (2018). TissueEnrich: Tissue-specific gene enrichment analysis. Bioinformatics, bty890, https://doi.org/10.1093/bioinformatics/bty890 | https://www.bioconductor.org/packages/release/bioc/html/TissueEnrich.html |
| tximeta R package (v1.24.0) | Michael I. Love, Charlotte Soneson, Peter F. Hickey, Lisa K. Johnson, N. Tessa Pierce, Lori Shepherd, Martin Morgan, Rob Patro Tximeta: Reference sequence checksums for provenance identification in RNA-seq PLOS Computational Biology 16(2): e1007664 | https://bioconductor.org/packages/release/bioc/html/tximeta.html |
| writexl R package (v1.5.1) | Ooms J (2024). writexl: Export Data Frames to Excel 'xlsx' Format. R package version 1.5.1, <https://CRAN.R-project.org/package=writexl>. | https://cran.r-project.org/package=writexl |
| **Other** | | |
| Human reference genome NCBI build 37, GRCh37 | Genome Reference Consortium | http://www.ncbi.nlm.nih.gov/projects/genome/assembly/grc/human/ |

Methods

Cellular models and cell and differentiation

Human pluripotent stem cell lines. Human induced pluripotent stem cell (iPSC) line ChiPSC18 (32-year-old; male) was purchased from Cellartis (Takara Bio Europe, Cat. No. Y00300), stored in liquid nitrogen gas phase at -150°C (~3x10^6^ cells per vial) and cultured in the Cellartis® DEF-CS^TM^ 500 culture system according to manufacturer’s instructions (Takara Bio Europe, Cat. No. Y30010). In brief, cell culture vessels were coated with 0.1 mL/cm^2^ DEF-CS COAT-1 solution diluted 1:20 in PBS with Ca^2+^ and Mg^2+^ and incubated at 37°C for at least 20 min. iPSC were thawed in a water bath at 37°C and placed into 4 mL of DEF-CS medium with all three additives (GF-1 (1:333), GF-2 (1:1000) and GF-3 (1:1000)). The cell suspension was centrifuged for 5 min at 300 g, supernatant was discarded and cells were resuspended in 0.2 mL/cm^2^ fresh DEF-CS medium with all three additives. Before transferring the cell suspension into the cell culture vessel, the coating solution was aspirated. Cells were placed in an incubator at 37°C and 5 % CO2. Medium was changed daily using DEF-CS medium containing only GF-1 (1:333) and GF-2 (1:1000). Cell cultures were inspected daily by light microscopy for confluency and spontaneous differentiation. Upon reaching a confluency of approximately 2-3x10^5^ cells/cm², cells were passaged. New cell culture vessels were coated as described above. The cells were washed with warm PBS without Ca^2+^ and Mg^2+^ and then incubated with 0.015 mL/cm^2^ of room-tempered TrypLE Select (ThermoFisher Scientific) at 37°C and 5 % CO2 for about 5 min. As soon as the cells lost contact to the cell culture vessel and the surrounding cells, the single cell suspension was resuspended with nine volumes of warm DEF-CS medium containing GF1-3. For passaging, 33,333 cells/cm^2^ were seeded in 0.2 mL/cm^2^ DEF-CS medium with all three additives into the newly prepared cell culture vessels. For further details, refer to Cellartis® DEF-CS^TM^ 500 culture system (Takara Bio Europe, Cat. No. Y30010). Cells used in this project were quality controlled by Takara Bio Europe to certify a normal karyotype and expression of stemness markers.

Primary Human Hepatocytes. Cryopreserved primary human hepatocytes (PHH) were purchased from BioIVT. Four different donors of PHH (three male, one female; all anonymous donor information is available) were used for RNA sequencing and qRT-PCR, and one donor (male) was used for immunofluorescence staining and bile canalicular excretion analysis. Cryopreserved PHH were cultured according to a published standard operating procedure (1) with minor modifications. In brief, 10mg of lyophilized rat-tail collagen (Roche Diagnostics) was dissolved in 40mL 0.2 % acetic acid (0.25mg/mL) at 4°C overnight. A standard cell culture 12-well plate (Sarstedt) was coated with 1mL/well of the prepared collagen solution. Then, the solution was aspirated and the plates were left to dry overnight under sterile conditions. PHH were thawed in a water bath at 37°C and transferred into 5mL culture medium (Williams E, 1% Penicillin-Streptomycin, 10µg/mL gentamycin, 10ng/mL insulin-transferrin-selenium (ITS), 2mM L-glutamine, 100nM dexamethasone and 10% fetal calf serum). Cells were centrifuged for 10min at 100g and resuspended in 10mL fresh culture medium. Before seeding, prepared cell culture plates were washed three times with PBS. Approximately 0.5x10^6^ cells (0.137x10^6^/cm^2^) were seeded in 1mL culture medium per well and then incubated for at least 3h at 37°C and 5% CO2. After initial incubation, the cells were washed three times with warm PBS. Additional 10mg of lyophilized rat-tail collagen was dissolved in 10mL of 0.2% acetic acid (1mg/mL solution). For each well, 37.25 µg/cm^2^ of the collagen solution were mixed with an appropriate amount of serum-free culture medium (without fetal calf serum) to reach a total volume of 2mL per 12-well and was added to each well on top of the seeded cells. Serum-free culture medium was changed every other day until further use.

Caco2 cells. Caco2 cells were purchased from ATCC. Cryopreserved cells were thawed in a 37°C water bath and transferred into 5 mL of culture medium (Dulbecco’s modified eagle medium, 20% fetal calf serum and 1% penicillin-streptomycin). Cells were centrifuged for 5 min at 300g and resuspended in fresh medium. In a T25 cell culture flask, 10,000 cells/cm^2^ were seeded in 15 mL medium and incubated at 37°C and 5% CO2. Medium was changed twice a week, and cells were passaged upon reaching 80 % confluency. For further analysis, Caco2 cells were seeded with a density of 50,000/cm^2^ in 24-well plates and differentiated for 21 days before fixation for immunofluorescence staining.

HepG2 cells. HepG2 cells were purchased from ATCC. Cryopreserved cells were thawed in a 37°C water bath and transferred into 5 mL of culture medium (Dulbecco’s modified eagle medium, 10% fetal calf serum and 1% penicillin-streptomycin). Cells were centrifuged at 300g for 5 min and resuspended in fresh culture medium. In a T75 cell culture flask 26,667 cells/cm^2^ were seeded in 15 mL culture medium and incubated at 37°C and 5% CO2. Medium was changed every three days and cells were passaged every six days. For further Western blot analysis, 0.5x10^6^ HepG2 cells were pelleted and stored at -80°C until further.

Differentiation of definitive endoderm and hepatocyte-like cells. Differentiation of WT, *CDX2^+/-^* and *CDX2^-/-^* iPSC to hepatocyte-like cells (HLC) was performed using the Cellartis® Definitive Endoderm Differentiation Kit (Takara Bio Europe, Cat. No.: Y30035) and the Cellartis® Hepatocyte Differentiation Kit (Takara Bio Europe, Cat. No.: Y30050) according to the manufacturer’s instructions with some modifications. For differentiation of iPSC towards definitive endoderm (DE), cell culture vessels were coated with 0.1 mL/cm^2^ of the Definitive Endoderm Coating and incubated at RT for at least 30 min. Upon reaching a cell density of approximately 2-3x10^5^ cells/cm², iPSC were detached from the culture vessel as described above and counted. Then, 33,333 cells per cm^2^ of the destination vessel were centrifuged for 5 min at 300g and resuspended in 0.2 mL of Definitive Endoderm Differentiation Day 0 medium per cm^2^ of the destination vessel. The coating solution was aspirated and the cell suspension was transferred into the cell culture vessel and then incubated at 37°C and 5% CO2. The cells were differentiated to definitive endoderm for the next 7 days with daily medium changes (except day 5) according to the manufacturer’s protocol. On day 7, DE cells were reseeded to further differentiate to HLC. First, culture vessels were coated with 0.1 mL/cm^2^ Biolaminin 111 LN and Biolaminin 521 LN (both BioLamina) in a 3:1 ratio diluted 1:20 in PBS containing Ca^2+^ and Mg^2+^ and incubated at 37°C for 30 min. DE cells were washed with warm PBS without Ca^2+^ and Mg^2+^ and then incubated with 0.1 mL/cm^2^ TrypLE Select at 37°C for 3 min. The single cell suspension was collected in a tube and the cell culture vessel was rinsed with 0.1 mL/cm^2^ PBS containing 10% FBS which was also collected in the tube. The cells were counted and centrifuged for 5 min at 300g and resuspended in Thawing and Seeding Medium from the Cellartis® Hepatocyte Differentiation Kit. Coating solution was aspirated, cells were seeded in a density of 1.3x10^5^ cells/cm2 in 0.5 mL/cm^2^ and incubated at 37°C and 5% CO2. For the next 18 days, cells were differentiated towards HLC according to the manufacturer’s protocol. On day 25, cells were collected for downstream applications.

CRISPR-Cas9 gene editing

CDX2 knockout by CRISPR-Cas9. The vector for CRISPR-Cas9-guided knockout of *CDX2* (hereafter referred to as KO-vector, Figure S3) was designed to contain a single guide RNA sequence (5’-CCTCTCAGAGAGCCCCAGCGTGG) targeting exon 2 of the *CDX2* coding sequence under control of a U6 promoter, hCas9 under control of a CBh promoter and GFP as selection marker under control of a CMV promoter and purchased from VectorBuilder (VB211007-1083juq; vector map available in Figure S3G). Transfection of iPSC was performed with the 4D-Nucleofector® System (Lonza, Cat. No.: AAF-1003B (4D-Nucleofector® Core Unit) and AAF-1003X (4D-Nucleofector® X Unit)) using the P3 Primary Cell 4D-Nucleofector® X Kit S (Lonza, Cat. No.: V4XP-3032). For each Nucleofection® of iPSC, 2x10^5^ cells were resuspended in 20 µL of the P3 Nucleofector® Solution with Supplement containing 0.8 µg of the KO-vector. Protocol CB-150 was applied. Immediately after Nucleofection®, cells were replated onto one well of a 24-well plate coated with COAT-1 (DEF-CS culture system, diluted 1:20 in PBS containing Ca^2+^ and Mg^2+^) in DEF-CS medium containing all three additives (GF-1, GF-2 and GF-3, diluted 1:333, 1:1000 and 1:1000, respectively). Cells were examined 24h post Nucleofection® for GFP signal. After 48h single, GFP-positive cells were picked using the CellCelector™ Flex (Sartorius) and transferred into single wells of a 96-well plate pre-coated with DEF-CS COAT-1 (1:10) containing DEF-CS medium containing GF1-3. Single cells were clonally expanded in the DEF-CS containing GF1-3 for 14 days, then only GF-1 and GF-2 were used. Cells were passaged to a larger well size whenever reaching a cell density of approximately 2-3x10^5^ cells/cm² until ~10x10^6^ cells were harvested for cryopreservation and analysis. iPSC generated in this project were verified to display expression of stemness markers (POU5F1, NANOG and SSEA4) (Figure S3H).

Genomic cleavage detection in iPSCs-clones. Clonally expanded cells were analyzed to identify clones containing insertions and/or deletions (indel) within exon 2 of *CDX2* using the GeneArt® Genomic Cleavage Detection Kit (Life Technologies, Cat. No.: A24372) according to manufacturer’s instructions with some modification. In brief, 0.5x10^6^ cells of WT-iPSC and clonally expanded iPSC (from now on called KO-iPSC) were harvested and lysed with 50 µL Cell Lysis buffer and 2 µL Protein Degrader. The lysates were incubated on a thermocycler at 68°C for 15min, followed by 10 min at 95°C to extract cellular DNA. The DNA region of interest of the CDX2 gene was amplified by PCR (primer sequence: FWD 5’-GCAGTTCTCAGCCCTCACTT and REV 5’-GCATCCTCCTGCTTCAGTCT) using 2 µL of the cell lysate, 1 µL of each forward and reverse primer at a concentration of 10 µM , 25 µL of the AmpliTaq Gold 360 Master Mix and 21 µL water (PCR program: 10 min at 95°C, 40x cycles of 30s at 95°C, 30s at 50°C (Tm of primer pair) and 30s at 72°C, followed by 7min at 72°C). The PCR products were verified on a 2% agarose gel. For the cleavage assay, 2 µL of the KO-PCR-product were mixed with 2 µL of the WT-PCR-product, 1 µL Detection Buffer and 4 µL water. On a thermocycler, the mixture was incubated at 95°C for 5 min to denature double stranded DNA, followed by a cooling process (95°C-85°C for -2°C/s and 85°C-25°C for -0.1°C/s) to randomly reanneal DNA strands. Next, 1 µL of the Detection Enzyme was added to each sample (water to the negative control), followed by incubation at 37°C for 1 h. DNA cleavage was analyzed on a 2% agarose gel. The CDX2 target region of iPSC clones showing a positive result in the cleavage assay was sequenced and compared to WT (Microsynth, primer sequence: 5’-GCAGTTCTCAGCCCTCACTT). Successful knockout of *CDX2* was confirmed after differentiation of the clonally expanded iPSCs to hepatocyte-like cells (HLC) by immunofluorescence staining and western blot against CDX2 (Figure 2C).

Agarose gel electrophoresis. Agarose gel electrophoresis was performed to assess results of DNA cleavage assays of iPSC clones suspected to carry a small insertion or deletion (indel) in the CDX2 target sequence following the knockout procedure. For this, 1 g agarose (Carl Roth) was dissolved in 50 mL TAE buffer (40 mM Tris, 20 mM acetic acid and 1 mM EDTA) and heated up in a microwave until fully dissolved. After cooling to about 50°C, 0.5 µg/mL ethidium bromide (ThermoFisher Scientific) was added to the agarose solution, mixed and transferred into the gel casting stand. Samples were loaded onto the gel and electrophoresis was performed in TAE buffer at 120 V for 30 min. The gel was analyzed on a UV table.

Immunofluorescence staining and quantification

Immunofluorescence staining. If not specified, all steps were performed at room temperature. iPSC, HLC, PHH and Caco-2 were fixed using 0.2 mL/cm^2^ 4% paraformaldehyde (PFA) for 15 min at 37°C. The fixed cells were washed three times with PBS, membranes were permeabilized with 0.5% Triton-X-100 for 10 min on an orbital shaker (this step was skipped for transmembrane proteins DPPIV, IBAT, SI, SLC5A1, and SSEA4), washed three times for 5 min and blocked for 1 h with blocking solution containing 3% BSA and 0.1% Tween-20 in PBS on an orbital shaker. Subsequently, cells were incubated with the primary antibody diluted in antibody dilution solution containing 0.3% BSA and 0.1% Tween-20 overnight at 4°C. The next day, cells were washed three times for 5 min with PBS and incubated with the secondary antibody diluted in antibody dilution solution for 2 h under light protection on an orbital shaker. Secondary antibodies used were Alexa Fluor® 488 Donkey anti-Rabbit (1:100), Alexa Fluor® 488 Donkey anti-Mouse (1:100), Alexa Fluor® 488 Donkey anti-Goat (1:100), Cy3AffiniPure Donkey anti-Rabbit (1:100) and Cy3AffiniPure Donkey anti-Mouse (1:100) (all Jackson Immuno Research Laboratories). Cells were washed three times with PBS for 5 min. Nuclear and actin staining was performed using DAPI solution (1:5000 in PBS) and Rhodamine-Phalloidin (ThermoFisher Scientific, 1:250 in PBS), respectively, for 15 min under light protection. Cells were washed three times with PBS before imaging and storage with PBS at 4°C.

Quantification of CDX2, HHEX and PROX1. For quantification of CDX2, HHEX and PROX1, immunofluorescence images were analyzed. At least five images with 20x magnification per biological replicate (n = 3) of WT and *CDX2^-/-^* HLC were captured resulting in a minimum of 15 fields of view per antibody and condition. Cell counts were determined at the hand of DAPI signal. Using ImageJ, a threshold for either the antibody or the DAPI stain using the default method was applied generating binary images. Two or more particles recognized as one were separated using the ImageJ built in watershed algorithm. The number of positive particles per antibody or stain was measured by counting each particle larger than 20 µm^2^ and smaller than 200 µm^2^. The percentage of positive cells was calculated by dividing the number of positive cells for the respective antibody by the number of counted nuclei per image. For CDX2 17 fields of view for each, WT-HLC and *CDX2^-/-^* HLC, for HHEX 20 fields of view for WT-HLC and 19 for *CDX2^-/-^* HLC, and for PROX1 19 fields of view for WT-HLC and 20 fields of view for *CDX2^-/-^* HLC were analyzed. The quantification raw data is available in Dataset S3.

Western blot

Protein isolation. Protein was isolated from cell pellets (~0.5x10^6^ cells) using 150 µL ice-cold RIPA buffer (50 mM TRIS-HCl, 150 mM NaCl, 1 % Nonidet P-40, 0.5 % sodium deoxycholate, 0.1 % SDS) containing protease inhibitor cocktail, phosphatase inhibitor I and phosphatase inhibitor II (all 1:100). Lysates were incubated for 20 min at 4°C, sonicated (50 % amplitude, intervals of 5 s pulse and 2 s break for 30 s) and centrifuged at 13,000 rpm at 4°C for 10 min. Supernatants containing protein were transferred into new tubes and stored at -80°C. Protein concentration was determined colorimetrically using the Pierce™ BCA Protein Assay Kit (ThermoFisher, Cat. No.: 23225) according to manufacturer’s instructions. Nine BSA dilutions ranging from 0 to 2000 µg/mL were used as a standard curve.

Protein gel electrophoresis. Gels were purchased from Bio-Rad (10 % Mini-PROTEAN® TGX Stain-FreeTM Protein Gels, 10-well comb, 50 µL, Cat. No.: #4568034). 30 µg of protein was mixed with 6 µL loading buffer (BioRad) and filled up to a total volume of 30 µL using water. Samples were incubated at 95°C for 5 min and loaded onto the gel. Used markers were MagicMark™ XP (ThermoFisher Scientific, Cat. No.: LC5602) and Precision Plus protein marker (Bio-Rad, Cat. No.: 1610374). Gel electrophoresis was performed at 100 V for 5 min, following 150 V for ~40 min until the dye front had moved out of the gel.

Blotting and immunolabeling. Separated proteins were transferred onto PVDF membranes for 40 min at 250 mA per gel using a semidry approach with the Trans-Blot® SD Semi-Dry Transfer Cell (BioRad), ROTI®Blot A and ROTI®Blot K as the anode and cathode buffer, respectively. After transfer, membranes were washed in TBST and blocked in 5 % BSA solution for 1 h on an orbital shaker at room temperature. Subsequently, membranes were incubated with primary antibody solution (anti-CDX2 (abcam, Cat. No.: ab76541) 1:2500 in 5 % BSA) at 4°C overnight on an orbital shaker. The next day, membranes were washed six times for 5 min in TBST and then incubated in secondary antibody solution (anti-rabbit-HRP 1:1000 (Cell Signaling Technologies, Cat. No.: 7074) in 5 % BSA) for 1 h. Membranes were washed again six times and then imaged using PURECLTM Dura HRP substrate (Vilber) according to manufacturer’s instructions.

qRT-PCR

RNA isolation and cDNA synthesis. Total RNA was collected as described previously (2). RNA was isolated from ChiPSC18 (WT-iPSC, D0, n = 4), ChiPSC18 *CDX2^+/-^* clone26 (*CDX2^+/-^* iPSC, D0, n=4), ChiPSC18 *CDX2^-/-^* clone2 (*CDX2^-/-^* iPSC, D0, n = 4), ChiPSC18-derived HLC (WT-HLC, D25, n = 5), ChiPSC18 *CDX2^+/-^* clone26-derived HLC (*CDX2^+/-^* HLC, D25, n = 4), ChiPSC18 *CDX2^-/-^* clone2-derived HLC (*CDX2^-/-^* HLC, D25, n = 6) and PHH (D0, n = 4). In brief, cells were collected and lysed in 1 ml Qiazol (Qiagen) and sonicated (50 % amplitude, intervals of 5 s pulse and 2 s break for 30 s). Then, 200 µL chloroform was added to each sample, shook thoroughly and centrifuged at 12,000 rpm for 15 min. The upper phase, containing the RNA, was transferred into 500 µL isopropanol without disturbing the lower phase (containing protein) or the interphase (containing DNA). After inverting the tube a few times, the sample was centrifuged again at 12,000 rpm for 15 min. Supernatant was discarded and the pellet was then washed three times with ethanol with decreasing concentrations (first absolute, 80 % and 70 %, respectively). The pellet was air dried for about 5 min until transparent and then resuspended in about 30 µL DEPC-treated water (ThermoFisher Scientific). RNA concentration, A260/280 and A260/230 values were measured using a NanoDropTM One (ThermoFisher Scientific, Cat. No.: ND-ONE-W). For cDNA synthesis, 2 µg of RNA was transcribed reversely using a high-capacity cDNA Reverse Transcription Kit (ThermoFisher Scientific, Cat. No.: 4368814) according to manufacturer’s instructions.

Quantitative real-time PCR (qRT-PCR) was performed using 25 ng of cDNA, TaqMan Universal Master Mix (ThermoFisher Scientific, Cat. No.: 4304437) and the specific TaqMan Assays from ThermoFisher Scientific. Each reaction was performed under the following conditions: 50°C for 2 min, 95°C for 10 min, followed by 40 cycles of 15 s at 95°C and 1 min at 60°C on a 7500 Real-Time PCR System (Applied Biosystems). The 2^−ΔΔCt^ method was applied, using GAPDH as a housekeeping gene and WT-iPSC as a reference population for normalizing expression levels.

RNA sequencing

RNA isolation and clean up. Total RNA was collected as described above. For further purification, extracted RNA was cleaned up with the RNeasy® Mini Kit (Qiagen, Cat. No.: 74104) according to the manufacturer’s instructions, including a DNase I digestion step. RNA concentration, A260/280 and A260/230 values were measured using the NanoDropTM One (ThermoFisher Scientific, Cat. No.: ND-ONE-W). For RNA Sequencing, four biological replicates of each WT-iPSC, CDX2^-/-^ iPSC, WT-HLC, *CDX2^-/-^* HLC and PHH were analyzed.

RNA-seq library preparation. RNA integrity was assessed on a 2100 Bioanalyzer with the RNA 6000 Nano Kit (Agilent Technologies) (RNA Integrity Number, RIN; range: 6.9-9.8). RNA concentrations were measured on a Qubit 4 Fluorometer with the RNA BR Assay Kit (Thermo Fisher). Sequencing libraries were generated from 500 ng RNA, using the TruSeq® Stranded mRNA Library Prep Kit (Illumina, Cat. No.: 20020595) with unique dual indexes (Illumina, Cat. No.: 20022371) according to the manufacturer’s instructions. Quantification of the libraries was performed with the Qubit 1X dsDNA HS Assay Kit (Thermo Fisher), and library sizes were checked on an Agilent 2100 Bioanalyzer with the DNA 1000 Kit (Agilent Technologies).

Next Generation Sequencing (NGS). The final libraries were normalized, pooled, diluted to 1.05 pM, and paired-end sequenced (2x75 bp) on a NextSeq 550 (Illumina) using the 500/550 High Output Kit v2.5 (150 cycles) kit (Illumina, Cat. No.: 20024907). 20 samples were analyzed in one sequencing run.

RNA-seq data analysis. RNA-seq data analysis was performed in R unless indicated otherwise. Mapping and quantification of the FASTQ files was done using Salmon (3) and the option ‘partial alignment’ with the online provided decoy-aware index for the mouse genome. To summarize the transcript reads on the gene level, the R package tximeta (4) (v1.16.1) was used. Gene-level transcript counts were filtered to exclude genes with less than 10 counts across all samples. Size factors for normalization were calculated using the function ‘estimateSizeFactors’ from the R package DESeq2 (5) (v1.44.0). Correlation among biological replicates was assessed using the R package PerformanceAnalytics (6) (v2.0.4) and biological replicates showing a Pearson correlation coefficient of more than 0.8 for iPSC and iPSC-derived cells and 0.7 for primary human hepatocytes from 4 different donors were accepted in the analysis. To obtain normalized expression values for analysis of gene expression variance, variance stabilizing transformation was performed using the function ‘vst’ from DESeq2 preceding principal component analysis (PCA). For PCA, a modified version of the ‘plotPCA’ function of DESeq2 was used to analyze a sequence of most variably expressed genes (100-2000 in 100 gene intervals) and principal components (1-5) to assess influential factors among cell type, differentiation stage, biological replicate, treatment and sequencing batch. Representative results were plotted using ggplot2 (7) (v3.5.1) for display in Figure 3A. Gene level log2 normalized counts were obtained from DESeq2 using the ‘counts’ function with ‘normalized’ set ‘TRUE’ and adding 0.5 before log2 transformation. ENSEMBL identifiers were annotated with official gene symbols using the package biomaRt (8) (v2.60.0) with the ‘hsapiens_gene_ensembl’ dataset. Absolute expression was plotted using ggplot2 with a boxplot geom and significant differences were annotated using the package ggsignif (9) with the t-test statistic and p-value annotation ‘*’=0.1 – ‘****’=0.0001. Differential gene expression was determined using the ‘DESeq’ function from DESeq2 with PHH as reference for robust dispersion estimation. Differentially expressed genes were clustered according to a supervised, logical differentiation pattern clustering procedure (DiPaC), previously described in Nell et. al 2022. Therefore, differentiation pattern groups (DPG) were derived from a set of three cut-off values for log2 fold changes that refer to changes in expression from start (e.g. expression values in iPSC) to end of a differentiation procedure (e.g. expression values in HLC) relative to a reference (e.g. PHH) (Figure 1D), or to differentiation states before (e.g. WT HLC) and after treatment (e.g. *CDX2^-/-^* HLC) relative to a reference (Figure 3C). The cut-offs were visualized as dotted lines in Figures 1D and 3C. Cut-off1 and 2 (vertical and horizontal dotted lines, respectively) were defined as the absolute log2 fold change of gene expression in population1 (x-axis) or population2 (y-axis), respectively, that discriminates expression levels similar (absolute log2 fold change < cut-off1) or dissimilar (absolute log2 fold change > cut-off1) to the reference (here PHH). Cut-off 3 (diagonal dotted lines) was defined as the minimum absolute difference in log2 fold changes of population1 and population2 representing a highly significant change in gene expression between both populations compared to the reference. For the analysis presented, all cut-off values were set to ‘2’. A detailed description can be found in the supplemental methods of Nell et al. 2022. This way, DiPaC generates differentiation pattern groups (DPG) containing genes with similar, biologically interpretable gene expression behavior such as DPG3 - ‘favorable upregulation’ or DPG5 - ‘adverse upregulation’ relative to the reference population. The code for performing DiPaC and corresponding downstream analysis is available from the lead contact. Enrichment analysis for tissue-associated gene expression according to the human protein atlas was performed using the R package TissueEnrich (10) (v1.24.1), with default parameters except of the ‘type’ argument, which was set to ‘4’ to obtain results for tissue group enriched genes. TissueEnrich results were visualized using the R package pheatmap (11) (v1.0.12). Gene ontology overrepresentation was analyzed using the R package clusterProfiler (12) (v4.12.0), with the ‘ont’ argument set to ‘BP’ for analysis of overrepresented GO terms related to biological processes. For transcription factor overrepresentation analysis, the R package enrichR (13) (v3.2) was used with the following datasets of TF-target gene associations: ‘ARCHS4_TFs_Coexp’, ‘Enrichr_Submissions_TF-Gene_Coocurrence’, ‘TF_Perturbations_Followed_by_Expression’, ‘TF-LOF_Expression_from_GEO’, ‘ENCODE_TF_ChIP-seq_2015’, ‘ENCODE_TF_ChIP-seq_2014’, ‘ENCODE_and_ChEA_Consensus_TFs_from_ChIP-X’, ‘ChEA_2013’, ‘ChEA_2015’, ‘Rummagene_transcription_factors’, available from the libraries section of the enrichR webpage hosted by the Ma’ayan laboratory. The function ‘enrichr’ was used to calculate TF overrepresentation estimates per DPG and all indicated datasets. Then, the expression change of all unique targets per TF and DPG was calculated, whereby the expression change of each target was defined as absolute of the log2 fold change of one population over PHH – log2 fold change of the other population over PHH. To obtain a weighted estimate of the influence of a transcription factor and its network on the gene expression changes observed per DPG, the GRN impact score was defined as the expression change score multiplied by the negative log10 of the p-Value associated with the enrichment of the corresponding transcription factor. In addition, RNA-seq based CellNet analysis was performed to assess establishment of cell type specific GRN establishment in engineered cells using the web application ‘PACNet’ (14) provided by the Cahan lab (http://ec2-44-201-176-192.compute-1.amazonaws.com/PACNet/webApp/) according to the recommended instructions and target cell-type set to ‘liver’ to obtain cell type classification results. All generated results are available in Dataset S1 (corresponding to Figure 1) and Dataset S4 (corresponding to Figure 3).

Comparisons of colon tissue with hiPSC, HLC and PHH. The collection of clinical specimens of human colorectal cancer patients for RNA sequencing has been described before (23). For the current study, four micro-dissected normal colon tissues of anonymized patients were employed. Total RNA was extracted from colon tissue dounced in 1 ml TRIzol (ThermoFisher) using the Direct-zol RNA MiniPrep Plus Kit (Zymo Research) including DNaseI treatment. Next, 200 ng total RNA were used for full-lenght reverse transcription with 5 cycles of pre-amplification. Libraries were prepared from cDNA using the Nextera DNA Library Prep Kit (Illumina) with 8 cycles of enrichment PCR followed by 0.9 × Ampure XP Beads (Beckman Coulter) purification.

Libraries were sequenced on a HiSeq2500 (Illumina) using TruSeq SBS Kit v3 – HS Chemistry in single read runs with read lengths of 94 bp. Reads were trimmed using Trim Galore! (v0.4.2, http://www.bioinformatics.babraham.ac.uk/projects/trim_galore/) to remove 3’ ends with base quality below 20 and adapter sequences. Reads were aligned to GRCh38 using STAR with per sample 2-pass mapping strategy (https://doi.org/10.1093/bioinformatics/bts635). PCR duplicates were detected using MarkDuplicate from Picard tools (version 1.115; http://broadinstitute.github.io/picard/). Gene-wise read counts were estimated based on Gencode release 30 (GRCh38.p12) using RSEM (https://doi.org/10.1201/b16589). Gene-level transcript counts were filtered to exclude genes with less than 10 counts across all samples. Size factors for normalization were calculated using the function ‘estimateSizeFactors’ from the R package DESeq2 (5) (v1.44.0). Gene level log2 normalized counts were obtained from DESeq2 using the ‘counts’ function with ‘normalized’ set ‘TRUE’ and adding 0.5 before log2 transformation. Colon tissue transcriptome data was provided as raw counts to guarantee donor anonymization (Dataset S6).’

Transcriptome hybrid state analysis of published protocols. Raw count data published in Ardisasmita et al 2022 was used as input for the comparison (15). Then, R Package Deseq2 was applied for calculation of scaling factors and robust estimation of dispersion in order to detect differentially expressed genes in four independent iPSC to hepatocyte protocols. Differentially expressed genes were then used in DiPaC with a cutoff of 1.5-fold change (log2), otherwise performed as described above to acquire differential pattern groups for all protocols. Superclusters of excessive and adverse upregulation were formed by merging gene lists of DPG4 and DPG5 for each protocol. Overlap between three and two independent protocols was created by merging gene lists of DPG4 and DPG5 superclusters for three protocols (Mun (16), Koui (17), Wang (18)) and gene lists of DPG4 and DPG5 superclusters for two further protocols (own protocol indicated as Cel, Gao (19)). Then, the overlap was visualized in Figure S2A by using R package VennDiagram. DPG4 and 5 genes according to DiPaC analysis for all protocols can be found in Dataset S4. Single cell sequencing data of 3D human liver organoids (Figure S2D) was obtained from the GSM4196642 sample in GSE141183 published in Shinozawa et al. 2021 (20) and analyzed in the Loupe browser v8.1.2 (10x Genomics) for visualization of co-expression (Log2 scale option) of HLC and IEC marker genes: HLC genes: *ALB, AFP, BAAT, TTR, F2, SERPINA7, APOA2, FGA, FGB, A1BG*; IEC genes: *CDX2, ISX, LCT, SI, ALPI, MUC13, MUC17, MEP1A, HEPH, MLN*.

Functional Assays

Lactase Activity Assay. The activity of lactase was tested with a Lactase Activity Assay Kit (Elabscience, Cat. No.: E-BC-K131-M) according to manufacturer’s instructions with minor changes. In brief, WT and *CDX2^-/-^* HLC were washed three times with PBS and the enzyme substrate (lactose) was added directly into the cell culture well. Supernatant was collected after 20 min, 40 min, 60 min, 120 min and 180 min of incubation. 8 µL of the supernatant were mixed with 200 µL of chromogenic agent solution (containing a non-specified glucose enzyme, peroxidase, phenol and a chromogenic substrate). Lactase cleaves lactose and produces glucose which in turn is catalyzed producing hydrogen peroxide. In the presence of hydrogen peroxide and phenol the peroxidase turns the chromogenic substrate into a red product. Absorption was measured spectroscopically using a plate reader at 505 nm and was proportional to glucose concentration and therefore to the activity of lactase.

Bile canalicular excretion assay. On day 25 of HLC differentiation, 5-chloromethylfluorescein (5-CMF) secretion into bile canalicular structures was examined in WT-HLC, *CDX2^-/-^* HLC and PHH as described previously (2) with minor changes. After 25 days of differentiation, HLCs cultured in 35 mm µ-dishes (ibidi, Cat. No.: 80136) were loaded onto a Zeiss LSM 880 confocal imaging system and treated with 10 µM 5-chloromethylfluorescein diacetate (CMFDA). PHH were treated the same way on day 3 after seeding. Imaging was started immediately after the addition of the substrate to avoid any delay. Excitation wavelength was set to 488 nm and emission was measured at 525 nm. For each, WT-HLC, *CDX2^-/-^* HLC and PHH, three biological replicates were imaged using a time-series of at least 20 images with an interval time of 3 min. For each replicate, five fields of view with three z-planes were captured. CMF fluorescence accumulation in canaliculi-like structures was analyzed by defining at least five regions of interest (ROIs) over these structures in captured time-lapse movies and measuring the change in mean fluorescence intensity over time in the ROIs. To account for disruption of canalicular membranes during the assay, supervised curation of data was applied to ensure that only the monotonically increasing phase was modelled. Data points were removed if their values were lower than the mean of the three preceding values minus twice their standard deviation, along with all subsequent measurements. Then, each temporal mean intensity profile was fitted independently to a three-parameter Gompertz model using the R package ‘drc’ (21) (version 3.0-1) to derive the maximum amplitude and the half time at each ROI. For a timepoint x and the parameters b, d and e, the mean intensity is given by:

$$\boldsymbol{f}\left( \boldsymbol{x} \right)\mathbf{=0+}\left( \boldsymbol{d}\mathbf{-0} \right)\left( \exp\left( \mathbf{-}\exp\left( \boldsymbol{b}\left( \boldsymbol{x}\mathbf{-}\boldsymbol{e} \right) \right) \right) \right)$$

The amplitude d describes the amount of CMF that is exported into canaliculi-like structures before the transport is saturated and the time constant describes the rate of CMF export, calculated as the time when the mean intensity reaches 50% of the amplitude. Corresponding raw data can be found in Dataset S5.

Quantification and statistical analysis

Statistical analyses for qPCR data, RNAseq data, quantification of CDX2, HHEX and PROX1 in immunofluorescence stainings and the lactase activity assay were performed using Student’s t-test. Differences among gene numbers in DiPaC superclusters were calculated using an exact binomial test from the R package ‘stats’. Odds ratio for overrepresentation of CDX2-associated genes was calculated by Fisher’s exact test. For the statistical analysis of the bile canalicular excretion assay a Wilcoxon signed-rank test was performed. Significance levels are indicated as *P<0.05, **P<0.01, ***P<0.001 and ****P<0.0001. The number of biological replicates shown in the figures is indicated in the figure legends as n = x biological replicates per group.

Figure S1: Comparison of absolute RNA transcript levels of selected genes from Figures 1-4 to transcript levels in primary colon tissue, as obtained by RNA sequencing. Expression levels are indicated as normalized counts (log2). Levels of significance: *: p=<0.05 - ****: p<0.0001.

Figure S2. The HLC-IEC hybrid state occurs in published and frequently applied 2D hepatocyte-like cell and human liver organoid (HLO) differentiation protocols. A) Venn diagram illustrating the overlap of DPG4 and DPG5 genes (excessive and adverse gene upregulation; respectively; corresponding to Figure 1C) obtained from genes in the intersection of several published 2D HLC differentiation protocols as obtained by DiPaC analysis of sequencing data published by Ardisasmita et al. 2022 ((GSE214097) and the protocol used in the present study (red: intersection of DPG4 and 5 genes of KOUI, WANG, CEL; blue: intersection of DPG4 and 5 genes from Gao, Mun) . B) Tissue group enrichment analysis of the union of A) (64 genes). The color bar indicates the number of unique tissue group enriched genes per tissue. C) Transcription factor overrepresentation in the union of A) (64 genes). The color bar indicates the number of unique target genes among all TF-target gene association datasets in the analysis. D) Single cell sequencing data of 3D human liver organoids as obtained from the GSM4196642 sample in GSE141183 published in Shinozawa et al. 2021 and analyzed in the Loupe browser v8.1.2 (10x Genomics) for visualization of co-expression of HLC and IEC marker gene lists as indicated (text box).

Figure S3. CRISPR Cas gene editing workflow to produce knock-out (KO) cells. A) Schematic of the gene editing process. Target cells are transfected with a plasmid encoding the Cas9 nuclease protein, a single-guide RNA (sgRNA) expression construct and EGFP to help identification of successfully transfected cells. Inside the cell, sgRNA and Cas9 form ribonucleoprotein (RNP) complexes that bind target DNA based on sgRNA and target DNA complementarity. Once bound to the target region (often upstream in the exon1 coding region), the endonuclease activity of Cas9 introduces double strand breaks (DSB) near the protospacer-adjacent motif (PAM) in the target region. The majority of DSB are repaired by non- homologous end-joining, which is error prone and likely to introduce small insertions or deletions (INDEL) in the target region. In some cases, the introduction of INDELS into the target region will result in a frameshift in the coding sequence and introduction of a pre-mature stop codon. The gene product will therefore be a non-functional, truncated protein, if the compromised mRNA is not degraded. B) Fluorescence micrograph of a single EGFP positive cell picked after CRIPSR Cas gene editing and seeded into a 96-well tissue culture plate for single cell cloning. C) Phase contrast micrograph of the expanded clone. D) Representative result of a T7 endonuclease-based heteroduplex cleavage assay. In brief, WT control and prospective KO DNA are denatured by heating respectively, and WT DNA molecules as well as a 1:1 suspension of WT and KO single strand molecules are allowed to re-anneal. Both reactions are then treated with a T7 endonuclease, which cuts heteroduplex DNA regions. In the event of a successful DSB introduction, followed by INDEL generation in the target coding region in a prospective KO clone, the annealing to WT DNA causes the formation of heteroduplex structures, which are cleaved during nuclease treatment. The results of the endonuclease treatment are analyzed by agarose gel electrophoresis to detect cleaved fragments (black arrows) in the WT: prospective KO DNA sample. E) DNA sequencing of the target region in prospective KO (magenta) and WT (green) cells. A representative example of a deletion in the target region close to the PAM sequence, which marks the binding and cleavage region of RNP complexes (blue), is shown. F) To confirm the success of the gene KO, expression of the target gene is investigated by western blotting. Here, a representative western blot is shown, where the absence of the gene product was confirmed in hepatocyte-like cells (HLC), which were derived from CRISPR Cas gene edited human induced pluripotent stem cells (iPSC), next to the colorectal carcinoma (CACO-2) and hepatoma (HepG2) cell lines, which served as additional controls. G) Vector map of the plasmid used for CDX2 knockout. H) Immunofluorescence images of pluripotency markers in *CDX2^-/-^* iPSC; from left to right: SSEA4, POU5F1, NANOG (green) and DAPI (blue).


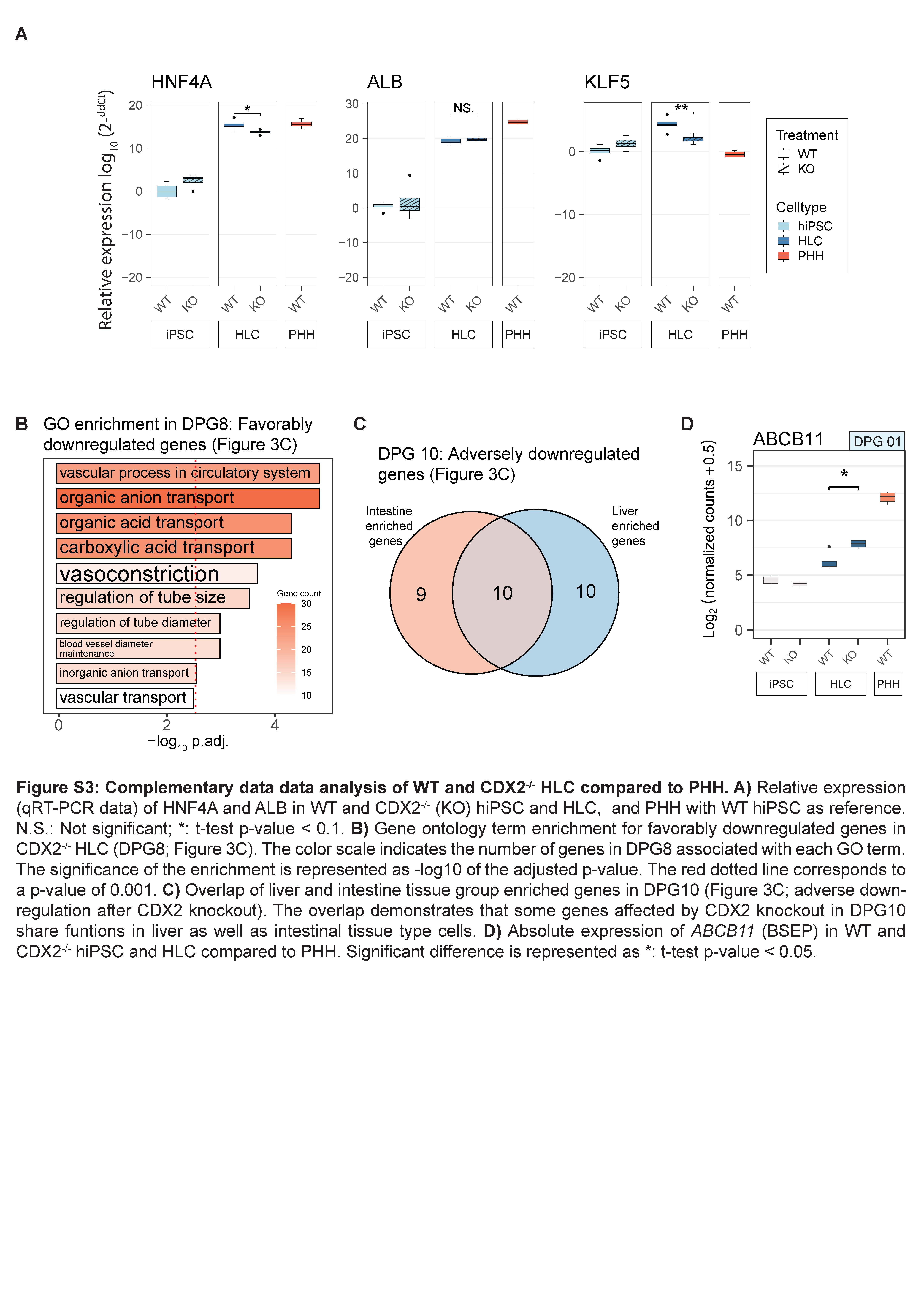


Figure S4. Complementary data data analysis of WT and CDX2^-/-^ HLC compared to PHH. A) Relative expression (qRT-PCR data) of HNF4A and ALB in WT and *CDX2^-/-^* (KO) iPSC and HLC, and PHH with WT iPSC as reference. N.S.: Not significant; *: t-test p-value < 0.1. B) Gene ontology term enrichment for favorably downregulated genes in *CDX2^-/-^* HLC (DPG8; Figure 3C). The color scale indicates the number of genes in DPG8 associated with each GO term. The significance of the enrichment is represented as -log10 of the adjusted p-value. The red dotted line corresponds to a p-value of 0.001. C) Overlap of liver and intestine tissue group enriched genes in DPG10 (Figure 3C; adverse downregulation after CDX2 knockout). The overlap demonstrates that some genes affected by CDX2 knockout in DPG10 share functions in liver as well as intestinal tissue type cells. D) Absolute expression of ABCB11 (BSEP) in WT and *CDX2^-/-^* iPSC and HLC compared to PHH. Significant difference is represented as *: t-test p-value < 0.05.


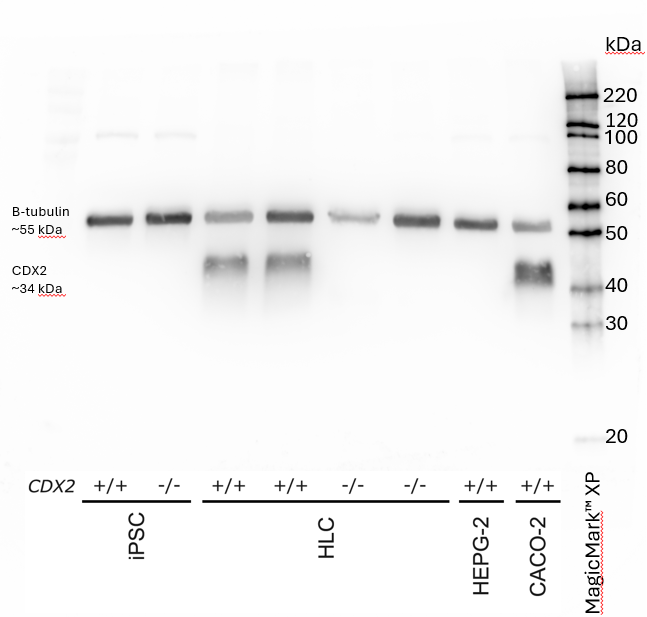


Figure S5. Western blot against CDX2 and beta-tubulin for WT and *CDX2^-/-^* iPSC and HLC, colorectal cancer cell line CACO-2 and hepatocellular carcinoma cell line HEPG2. The protein standard MagicMark™ XP indicates protein size in kDa.

**Figure S6:** Heterozygous knockout of *CDX2* is not sufficient to suppress intestinal differentiation signatures in HLC. A) DNA sequencing chromatogram of the Cas9 target region of *CDX2* exon 2 in a *CDX2^+/-^* knockout iPSC clone, indicating characteristic high background signal immediately downstream of the deletion in the target region (red arrow). The WT sequence is indicated below, with the deleted bases marked in red. B) Relative expression of hepatocyte (*ALB*, *HNF4A*), intestinal (*HNF4A*, *CDX2*, *KLF5*, *SI*, *HEPH*) and pluripotency markers (*POU5F1*, *NANOG*) in *CDX2^+/+^* (WT), *CDX2^+/-^* and *CDX2^-/-^* iPSC, HLC, and PHH for comparison (WT iPSC as the reference). Levels of significance (t-test): *: p=<0.05 - ****: p<0.0001; N.S.: not significant. C) Immuno-fluorescence imaging of *CDX2^+/-^* iPSC-derived HLC, stained for CDX2 (red), AFP (green) and DAPI (blue), illustrating a faint nuclear signal for CDX2 at 1 s of exposure time in the heterozygous clone.

Dataset S1 (separate file). DiPaC and downstream analysis corresponding to Figure 1. The dataset contains results of differential gene expression (DEG) analysis of WT iPSC and HLC compared to PHH (mean expression; log2 fold changes; p-values) as obtained by the DEseq2 analysis pipeline with annotations of their respective cluster association following DiPaC analysis for obtaining biologically interpretable differentiation pattern groups (DPG). Additional sheets contain results from tissue group enrichment, gene ontology overrepresentation and transcription factor overrepresentation analysis.

Dataset S2 (separate file). Hybrid differentiation genes obtained from published protocols and DiPaC analysis. For each analyzed protocol (Mun, Koui, Wang, Gao and Cel) DPG4 (excessively expressed genes) and DPG5 (adversely expressed genes) genes as obtained by Differentiation Pattern Clustering (DiPaC) based on gene expression in iPSC, HLC and PHH populations are listed, as well as unique hybrid genes of protocol combinations and the overlap of hybrid genes expressed by HLC of all protocols, as specified in the SI Materials and Methods section and corresponding to Figure S2 of the main manuscript.

Dataset S3 (separate file). Immunofluorescence quantification data. The dataset contains quantification data of positive immunofluorescence signals per field of view, experiment (name), stain (dye), protein targeted in the experiment (protein), number of positive nuclei (count), and identifiers for biological and technical replicates (BR and TR), as specified in the SI Materials and Methods section and corresponding to Figure 2E and D of the main manuscript.

Dataset S4 (separate file). DiPaC and downstream analysis corresponding to Figure 3. The dataset contains results of differential gene expression (DEG) analysis of WT and *CDX2^-/-^* iPSC and HLC compared to PHH (mean expression; log2 fold changes; p-values) as obtained by the DEseq2 analysis pipeline with annotations of their respective cluster association following DiPaC analysis for obtaining biologically interpretable differentiation pattern groups (DPG). Additional sheets contain results from tissue group enrichment, gene ontology overrepresentation, transcription factor overrepresentation and PACNet cell identity scoring analysis.

Dataset S5 (separate file). CMFDA assay data. The dataset contains time series fluorescence intensity measurements of CMF signals in bile canaliculi of WT and CDX2^-/-^ HLC, as well as PHH obtained by laser scanning microscopy with excitation at 488 nm and emission at 525 nm as specified in the SI Materials and Methods section. Timepoints are given in min (time). Experiment identifiers (diff), celltype (for HLC: WT or KO; PHH), field of view identifier, object type, object identifier, area of the measured object, mean intensity, min and max intensities are provided.

Dataset S6 (separate file). Raw counts corresponding to Figure S6. The dataset contains raw counts of RNA sequencing experiments of iPSC (n=4), PHH (n=3), colon tissue (n=4) and HLC (n=4) mapped to GRCh38 as reference.

**SI References**

1. X. Gu, *et al.*, Relevance of the incubation period in cytotoxicity testing with primary human hepatocytes. *Arch Toxicol* **92**, 3505–3515 (2018).

2. P. Nell, *et al.*, Identification of an FXR-modulated liver-intestine hybrid state in iPSC-derived hepatocyte-like cells. *J Hepatol* **77**, 1386–1398 (2022).

3. R. Patro, G. Duggal, M. I. Love, R. A. Irizarry, C. Kingsford, Salmon provides fast and bias-aware quantification of transcript expression. *Nature Methods 2017 14:4* **14**, 417–419 (2017).

4. M. I. Love, *et al.*, Tximeta: Reference sequence checksums for provenance identification in RNA-seq. *PLoS Comput Biol* **16**, e1007664 (2020).

5. M. I. Love, W. Huber, S. Anders, Moderated estimation of fold change and dispersion for RNA-seq data with DESeq2. *Genome Biology 2014 15:12* **15**, 1–21 (2014).

6. B. G. Peterson, P. Carl, Econometric Tools for Performance and Risk Analysis [R package PerformanceAnalytics version 2.0.8]. *CRAN: Contributed Packages* (2024). https://doi.org/10.32614/CRAN.PACKAGE.PERFORMANCEANALYTICS.

7. H. Wickham, *et al.*, Create Elegant Data Visualisations Using the Grammar of Graphics [R package ggplot2 version 3.5.2]. *CRAN: Contributed Packages* (2025). https://doi.org/10.32614/CRAN.PACKAGE.GGPLOT2.

8. D. Smedley, *et al.*, BioMart - Biological queries made easy. *BMC Genomics* **10**, 1–12 (2009).

9. C. Ahlmann-Eltze, I. Patil, Significance Brackets for “ggplot2” [R package ggsignif version 0.6.4]. *CRAN: Contributed Packages* (2022). https://doi.org/10.32614/CRAN.PACKAGE.GGSIGNIF.

10. A. Jain, G. Tuteja, TissueEnrich: Tissue-specific gene enrichment analysis. *Bioinformatics* **35**, 1966–1967 (2019).

11. pheatmap: Pretty Heatmaps. *CRAN: Contributed Packages* (2010). https://doi.org/10.32614/CRAN.PACKAGE.PHEATMAP.

12. T. Wu, *et al.*, clusterProfiler 4.0: A universal enrichment tool for interpreting omics data. *The Innovation* **2**, 100141 (2021).

13. M. V. Kuleshov, *et al.*, Enrichr: a comprehensive gene set enrichment analysis web server 2016 update. *Nucleic Acids Res* **44**, W90 (2016).

14. E. K. W. Lo, *et al.*, Platform-agnostic CellNet enables cross-study analysis of cell fate engineering protocols. *Stem Cell Reports* **18**, 1721–1742 (2023).

15. A. I. Ardisasmita, *et al.*, A comprehensive transcriptomic comparison of hepatocyte model systems improves selection of models for experimental use. *Communications Biology 2022 5:1* **5**, 1–15 (2022).

16. S. J. Mun, *et al.*, Generation of expandable human pluripotent stem cell-derived hepatocyte-like liver organoids. *J Hepatol* **71**, 970–985 (2019).

17. Y. Koui, *et al.*, An In Vitro Human Liver Model by iPSC-Derived Parenchymal and Non-parenchymal Cells. *Stem Cell Reports* **9**, 490 (2017).

18. Y. Wang, *et al.*, Defined and Scalable Generation of Hepatocyte-like Cells from Human Pluripotent Stem Cells. *J Vis Exp* **2017**, e55355 (2017).

19. X. Gao, *et al.*, Hepatocyte-like cells derived from human induced pluripotent stem cells using small molecules: Implications of a transcriptomic study. *Stem Cell Res Ther* **11**, 1–21 (2020).

20. T. Shinozawa, *et al.*, High-Fidelity Drug-Induced Liver Injury Screen Using Human Pluripotent Stem Cell–Derived Organoids. *Gastroenterology* **160**, 831-846.e10 (2021).

21. C. Ritz, F. Baty, J. C. Streibig, D. Gerhard, Dose-Response Analysis Using R. *PLoS One* **10**, e0146021 (2015).
